# *RFX6* haploinsufficiency predisposes to diabetes through impaired beta cell function

**DOI:** 10.1007/s00125-024-06163-y

**Published:** 2024-05-14

**Authors:** Hazem Ibrahim, Diego Balboa, Jonna Saarimäki-Vire, Hossam Montaser, Oleg Dyachok, Per-Eric Lund, Muhmmad Omar-Hmeadi, Jouni Kvist, Om P. Dwivedi, Väinö Lithovius, Tom Barsby, Vikash Chandra, Solja Eurola, Jarkko Ustinov, Tiinamaija Tuomi, Päivi J. Miettinen, Sebastian Barg, Anders Tengholm, Timo Otonkoski

**Affiliations:** 1 Stem Cells and Metabolism Research Program, Faculty of Medicine, University of Helsinki, Helsinki, Finland; 2 Department of Medical Cell Biology, Uppsala University, Uppsala, Sweden; 3 Institute for Molecular Medicine Finland, FIMM, HiLIFE, Helsinki, Finland; 4 Research Program of Clinical and Molecular Metabolism, University of Helsinki, Helsinki, Finland; 5 Folkhälsan Institute of Genetics, Folkhälsan Research Center, Biomedicum Helsinki, Finland; 6 Abdominal Center, Endocrinology, University of Helsinki and Helsinki University Hospital, Helsinki, Finland; 7 Lund University Diabetes Centre, Department of Clinical Sciences, Lund University, Lund, Sweden; 8 Department of Pediatrics, Helsinki University Hospital, Helsinki, Finland

**Keywords:** Beta cells, Glucose-stimulated insulin secretion, Isogenic allelic series models, Monogenic diabetes, Stem-cell-derived islets, Type 2 diabetes

## Abstract

**Aims/hypothesis:**

Regulatory factor X 6 (RFX6) is crucial for pancreatic endocrine development and differentiation. The *RFX6* variant p.His293LeufsTer7 is significantly enriched in the Finnish population, with almost 1:250 individuals as a carrier. Importantly, the FinnGen study indicates a high predisposition for heterozygous carriers to develop type 2 and gestational diabetes. However, the precise mechanism of this predisposition remains unknown.

**Methods:**

To understand the role of this variant in beta cell development and function, we used CRISPR technology to generate allelic series of pluripotent stem cells. We created two isogenic stem cell models: a human embryonic stem cell model; and a patient-derived stem cell model. Both were differentiated into pancreatic islet lineages (stem-cell-derived islets, SC-islets), followed by implantation in immunocompromised NOD-SCID-Gamma mice.

**Results:**

Stem cell models of the homozygous variant *RFX6*^−/−^ predictably failed to generate insulin-secreting pancreatic beta cells, mirroring the phenotype observed in Mitchell–Riley syndrome. Notably, at the pancreatic endocrine stage, there was an upregulation of precursor markers *NEUROG3* and *SOX9*, accompanied by increased apoptosis. Intriguingly, heterozygous *RFX6*^+/−^ SC-islets exhibited *RFX6* haploinsufficiency (54.2% reduction in protein expression), associated with reduced beta cell maturation markers, altered calcium signalling and impaired insulin secretion (62% and 54% reduction in basal and high glucose conditions, respectively). However, *RFX6* haploinsufficiency did not have an impact on beta cell number or insulin content. The reduced insulin secretion persisted after in vivo implantation in mice, aligning with the increased risk of variant carriers to develop diabetes.

**Conclusions/interpretation:**

Our allelic series isogenic SC-islet models represent a powerful tool to elucidate specific aetiologies of diabetes in humans, enabling the sensitive detection of aberrations in both beta cell development and function. We highlight the critical role of RFX6 in augmenting and maintaining the pancreatic progenitor pool, with an endocrine roadblock and increased cell death upon its loss. We demonstrate that *RFX6* haploinsufficiency does not affect beta cell number or insulin content but does impair function, predisposing heterozygous carriers of loss-of-function variants to diabetes.

**Data availability:**

Ultra-deep bulk RNA-seq data for pancreatic differentiation stages 3, 5 and 7 of H1 *RFX6* genotypes are deposited in the Gene Expression Omnibus database with accession code GSE234289. Original western blot images are deposited at Mendeley (https://data.mendeley.com/datasets/g75drr3mgw/2).

**Graphical Abstract:**

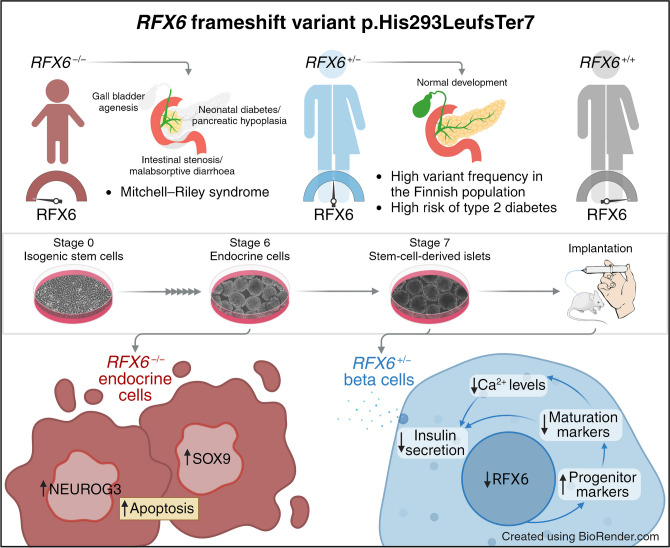

**Supplementary Information:**

The online version contains peer-reviewed but unedited supplementary material available at 10.1007/s00125-024-06163-y.


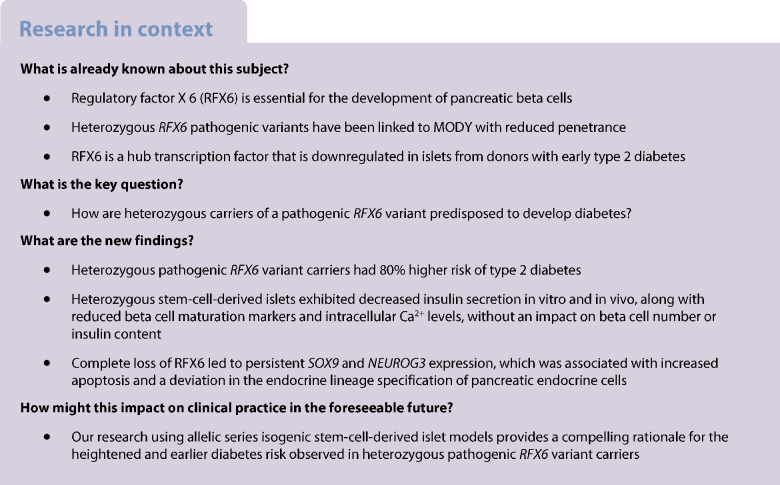


## Introduction

Diabetes is a metabolic disorder characterised by the inability of pancreatic beta cells to control blood glucose levels. The aetiology of the most common form, type 2 diabetes, is heterogeneous, although population genetic studies have identified numerous type 2 diabetes-associated genetic variants in loci of genes expressed in the beta cell [1, 2]. Pathogenic variants in these beta cell genes lead to monogenic diabetes, presenting either as neonatal diabetes or MODY [3]. Regulatory factor X 6 (RFX6) is a winged-helix transcription factor that regulates genes required for the development of the pancreas and other intestinal organs [4–7]. Autosomal recessive variants in *RFX6* cause Mitchell–Riley syndrome (OMIM, 615710; www.omim.org), characterised by intrauterine growth retardation, annular or hypoplastic pancreas, permanent neonatal diabetes, gall bladder hypoplasia or agenesis, intestinal stenosis with malabsorptive diarrhoea and, in some cases, pancreatic exocrine insufficiency [6, 8, 9]. Recently, a less severe manifestation of Mitchell–Riley syndrome was documented, involving compound heterozygous variants that are not fully inactivating. This condition presents as childhood-onset diabetes, typically occurring between the ages of 2 and 5 years [10, 11].

In mice, *Rfx6* is initially expressed in the definitive endoderm, before becoming progressively confined to the gut and dorsal pancreatic bud, and then islet progenitor cells where it is required for the differentiation of all islet cell types, except for pancreatic polypeptide (PP)-producing cells. Mice with loss of *Rfx6* exhibited similarities to the human phenotype, presenting with neonatal diabetes and intestinal obstruction, albeit with variable pancreatic hypoplasia [6]. While homozygous *Rfx6* mutant mice had severe symptoms and died shortly after birth, heterozygous mutants did not show signs of diabetes [6]. Conditional *Rfx6* ablation in adult mouse beta cells resulted in impaired glucose-stimulated insulin secretion (GSIS), without compromised beta cell mass or insulin content. The defective insulin secretion was attributed to the downregulation of beta cell maturation genes *Gck*, *Abcc8*, *Ucn3* and voltage-dependent calcium channel (VDCC) genes, concomitant with the upregulation of beta cell disallowed genes such as *Slc16a1*, *Ldha*, and *Igfbp4* [12]. Similarly, knockdown of *RFX6* in the human beta cell insulinoma line EndoC-βH2 resulted in reduced insulin gene transcription and defective GSIS through reducing VDCC gene expression [13]. A recent study also demonstrated that *RFX6* knockdown in primary human islets reduced GSIS to the level seen in islets from donors with type 2 diabetes, through transcriptionally dysregulated vesicle trafficking, exocytosis and ion transport pathways [14].

Heterozygous *RFX6* pathogenic variants have been linked to MODY with reduced penetrance in humans [15–21]. Moreover, genome-wide association studies have associated variants of *RFX6* with type 2 diabetes [22, 23]. In adult primary islets, RFX6 was shown to be a hub transcription factor that was downregulated in islets from donors with early type 2 diabetes, correlated with reduced GSIS [14]. Additionally, genetic variants that increase type 2 diabetes risk are predicted to disrupt RFX-binding motifs [24].

The precise mechanism of how heterozygous pathogenic *RFX6* variant carriers are predisposed to develop diabetes remains unknown. Therefore, we sought to use patient-derived and embryonic stem cells combined with CRISPR-based genetic engineering to create isogenic allelic series models of a specific *RFX6* frameshift variant, circumventing the use of unphysiological systems of complete gene knockout or knockdown. Human pluripotent stem cells have been extensively used to model monogenic diabetes genes [25], including *RFX6* [9, 26, 27], as they can be differentiated into stem-cell-derived islets (SC-islets) that closely mimic native human islets developmentally and functionally. Employing our optimised protocol to generate highly functional SC-islets [28, 29], we elucidate the impact of homozygous and heterozygous *RFX6* pathogenic variants on pancreatic endocrine development and beta cell function.

## Methods

### Lifetime risk analysis of type 2 and gestational diabetes in the FinnGen dataset

We analysed the risk of gestational or type 2 diabetes, the age at diagnosis of diabetes and BMI in the *RFX6* frameshift variant (p.His293LeufsTer7) carriers compared with non-carriers in the FinnGen data freeze R11 at individual level, using genome-wide genotype data and longitudinal healthcare registry data [23, 30]. FinnGen (https://www.finngen.fi/en) is a public–private research project, combining genome and digital healthcare data gathered since 2017. In FinnGen, diseases are defined as endpoints by FinnGen clinical expert teams according to ICD codes of versions 8 (1969–1986), 9 (1987–1995; http://www.icd9data.com/2007/Volume1/default.htm) and 10 (1996–2019; https://icd.who.int/browse10/2019/en). FinnGen R11 comprises 440,734 Finnish European individuals (44% male and 56% female) with informed consent for biobank research based on the Finnish Biobank Act, or regarding earlier cohorts, with approval from Fimea, the National Supervisory Authority for Welfare and Health. As the database is based in Finland, where the variant under study is enriched, the majority of samples belong to Finnish ethnicity, with no exclusions made based on gender or sex. The type 2 diabetes population comprised 55% male and 45% female individuals. The FinnGen study protocol (no. HUS/990/2017) is approved by the Coordinating Ethics Committee of the Hospital District of Helsinki and Uusimaa (HUS).

### Generating induced pluripotent stem cells from an individual with Mitchell–Riley syndrome, and stem cell culturing

Dermal fibroblasts obtained from a skin biopsy from an individual with homozygous *RFX6* frameshift variant at 6 months of age were reprogrammed using Sendai viruses (SeVdp), expressing *OCT4*, *SOX2*, *KLF4* and *C-MYC* reprogramming factors. The formed HEL118 induced pluripotent stem cell (iPSC) lines were verified using Sanger sequencing to confirm the frameshift variant, were tested negative for mycoplasma contamination and did not contain transgene vectors. Additionally, the iPSCs line HEL46.11 (derived from human neonatal foreskin fibroblast) and the human embryonic stem cell (hESC) line H1 (WA01, WiCell; Madison, USA), which are wild-type *RFX6*, were used in this study. All the generated stem cell lines were cultured on Matrigel-coated (Corning, no. 354277; USA) plates with E8 medium (Thermo Fisher Scientific, A1517001; USA) and passaged using 0.5 mmol/l EDTA (Life Technologies, 15575–038; USA) in PBS as a dissociation agent. The patient’s iPSCs were derived after informed consent from parents and according to the approval of the coordinating ethics committee of the Helsinki and Uusimaa Hospital District (no. 423/13/03/00/08). The patient’s clinical data were used after informed consent from parents.

### Genome editing of iPSCs and H1 hESCs

For correcting the *RFX6* frameshift variant in the patient-derived HEL118.3 iPSCs, we used a plasmid-based CRISPR–Cas9 system with a guide and a correction template that were designed with Benchling (Biology Software, 2017, https://benchling.com) (gRNA1 correction: ATAACAGGATTTTCGAGCAG). The correction template introduced a TseI restriction site by silent mutations to facilitate the screening of the edited clones. Two million iPSCs were electroporated with 6 µg of CAG-Cas9-T2A-EGFP-ires-Puro (Addgene, plasmid no. 78311), 500 ng of the gRNA cassette and 4 µg of the 200 bp dsDNA correction template, using Neon Transfection system (Thermo Fisher Scientific; 1100 V; 20 ms; two pulses). Single cells were sorted, expanded and screened using TseI restriction enzyme. The editing resulted in heterozygous clones only; one heterozygous clone was electroporated using gRNA2 correction guide (AGCAGGGGAAGGAGATGGTC) to obtain the homozygous clone. All clones (iPSCs; #*RFX6*^−/−^, *#RFX6*^+/−^ and *#RFX6*^+/+^) were further validated by Sanger sequencing.

For introducing the *RFX6* frameshift variant in H1 hESCs, we used a more efficient ribonucleoprotein (RNP) CRISPR–Cpf1 system (Integrated DNA Technologies, USA), with a guide and a mutation template that were designed with Benchling (Biology Software, 2017) (gRNA mutation: TTACACTTTTGGCAAGGAATG). Two million cells were electroporated with 10 µg Alt-R A.s. Cas12a (Cpf1) Ultra combined with the gRNA and 4 µg of the 100-nt ssODN correction template (Integrated DNA Technologies), using Neon Transfection system (Thermo Fisher Scientific; 1100 V; 20 ms; two pulses). Single cells were sorted, expanded and screened using TseI restriction enzyme. Since the cut-site is 13 nucleotides away from the mutation, the suboptimal editing resulted in heterozygous clones only. The heterozygous clone 3G was electroporated with the same gRNA and a mutation template carrying TseI and NheI restriction sites to facilitate the screening of homozygous clones. All clones (hESCs; *RFX6*^+/+^, *RFX6*^+/−^ 9C, *RFX6*^+/−^ 3G and *RFX6*^−/−^) were further validated by Sanger sequencing. The top three CRISPR off-target hits were sequenced and did not have any indels. Karyotype analyses based on chromosomal G-banding were performed for all the generated hiPSC and hESC clones at Ambar Lab, Barcelona, Spain. All cell lines tested negative for mycoplasma. Primer sequences used for genome editing are described in electronic supplementary material (ESM) Table 1.

### In-vitro pancreatic endocrine differentiation culture

HEL118.3 iPSCs were differentiated towards the pancreatic lineage to generate pancreatic endocrine cells using our previously published protocol with minor modifications [31]. Briefly, two million cells/well were seeded on six-well Matrigel-coated plates in E8 medium supplemented with 10 µmol/l Rho-Associated kinase inhibitor (ROCKi, Y-27632). The differentiation was started following 24 h of seeding and proceeded through a six-stage differentiation protocol in monolayer. The hESC H1 cell lines were differentiated using the extended seven-stage protocol (stages 1–7 [S1–S7]: S1–S4 in adherent culture; S5 in AggreWell [Stemcell Technologies, no. 34421; Vancouver, Canada] and S6 and S7 in suspension culture) [28, 29]. The detailed differentiation protocol and the stage-specific complete media formulations are described in ESM Tables 2, 3. The details of the reagents used in the differentiation protocol are described in ESM Table 4.

### Western blot

For protein extraction, cells were washed with ice-cold PBS and lysed with Cell lysis buffer (Cell Signalling Technology no. 9803; USA) for 10 min on ice. The cells were sonicated for 3×5 s on ice, centrifuged (10,000 *g* for 10 min at 4°C) and the supernatant fraction was stored at −80°C. The samples were run on Any kD Mini-PROTEAN TGX gel (Bio-Rad, Hercules, USA) and then dry-transferred onto a nitrocellulose membrane using the iBlot system (Invitrogen; USA) as per manufacturer’s instructions. The membrane was then probed with the primary antibody overnight at 4°C, washed twice with TBS (Medicago no. 09-7500; Uppsala, Sweden) containing 0.1% Tween for 2×10 min, and incubated with the corresponding secondary antibody for 30 min at room temperature. Chemiluminescence detection was performed with Amersham ECL (Cytiva, RPN2235; USA) and Bio-Rad Chemidoc XRS1 imaging system; Image Lab software v6.0.0 (Bio-Rad). Densitometry quantification analysis was done using Fiji software v1.53 [32]. The details of antibodies and their dilutions used for western blotting are described in ESM Table 5.

### Flow cytometry

To quantify the percentage of definitive endoderm-positive cells at stage 1, flow cytometry for C-X-C motif chemokine receptor 4 was performed on one million dissociated cells using TrypLE (Thermo Fisher Scientific) for 3 min at 37°C. Cells were resuspended in cold 5% vol./vol. FBS-containing PBS and the conjugated antibodies were added and incubated for 30 min at room temperature. For intracellular antigen cytometry of stages 4, 5, 6 and 7, cells were dissociated with TrypLE for 8 min at 37°C and resuspended in cold 5% FBS-containing PBS. A total of one million cells were fixed and permeabilised using Cytofix/Cytoperm (554714, BD Biosciences, USA) as per manufacturer’s instructions. Primary, conjugated or IgG isotype (negative control) antibodies were incubated with the cells overnight at 4°C in Perm/Wash buffer (554714, BD Biosciences) containing 4% vol./vol. FBS and then secondary antibodies for 45 min at room temperature. The cells were then analysed using a FACSCalibur cytometer (BD Biosciences) with BD Cellquest Pro v4.0.2 (BD Biosciences) and FlowJo software v9 (BD Biosciences). The details of antibodies and their dilutions for flow cytometry are given in ESM Table 5.

### Immunocytochemistry and immunohistochemistry

For whole mount or adherent culture staining, cells were fixed with 4% wt/vol. paraformaldehyde (PFA) for 15 min at room temperature, permeabilised with 0.5% vol./vol. Triton X-100 in PBS for 15 min at room temperature, then blocked with Ultra V block (Thermo Fisher Scientific) for 10 min followed by incubation with primary antibodies overnight at 4°C and secondary antibodies for 1 h at room temperature diluted in 0.1% vol./vol. Tween in PBS. For paraffin embedding, aggregates were fixed with 4% PFA at 4°C for 24 h then embedded in 2% low-melting agarose (Fisher Bioreagents; USA) PBS and transferred to paraffin blocks. Implanted grafts were retrieved after 3 months, dissected and fixed with 4% PFA at room temperature for 48 h before being paraffin embedded and cut into 5 µm sections using a Leica microtome. For immunohistochemistry, slides were deparaffinised and antigen-retrieved by boiling slides in 0.1 mol/l citrate buffer (pH 6) using a Decloaking chamber (Biocare Medical, USA) at 95°C for 12 min. For the TUNEL assay, an In Situ Cell Death Detection Kit, Fluorescein (no. 11684795910, Roche, Basel, Switzerland) was used according to the manufacturer’s instruction. EVOS FL Digital Inverted Fluorescence Microscope (Invitrogen, USA) or a Zeiss Axio Observer Z1 with Apotome (ZEN-2 software, Germany) were used for image acquisition. All samples were randomised, blinded, equally treated and acquired with the same microscope parameters. Image quantification was performed using CellProfiler software v4.2.1 [33], and Fiji software v1.53 [32]. The details of antibodies and their dilutions used in the study for immunofluorescence are described in ESM Table 5.

### RNA extraction and quantitative RT-PCR

Total RNA was extracted using a NucleoSpin Plus RNA kit (Macherey-Nagel, Germany). A total of 1.5 µg RNA was reversely transcribed using 0.5 μl Moloney murine leukaemia virus reverse transcriptase (Promega, M1701; USA), 4 µl RT buffer 5× (Promega), 2.5 μl dNTPs 2.5 mmol/l, 1 μl Oligo-dT 500 μg/ml (Promega), 0.2 μl Random hexamers 500 μg/ml (Promega) and 0.5 μl Riboblock RNase inhibitor 40 u/μl (Fermentas, USA) for 90 min at 37°C. The generated cDNA was amplified using 5× HOT FIREPol EvaGreen qPCR Mix Plus no ROX (Solisbiodyne, Estonia) in a 20 µl reaction. The reactions were pipetted using QIAgility (Qiagen, Germany) robot into a 100-well disc run in Rotor-Gene Q. Relative quantification of gene expression was analysed using the ${2}^{{-\Delta \Delta {\text{C}}}_{{\text{t}}}}$ method, with cyclophilin G (*PPIG*) as a reference gene. Reverse transcription without template was used as a negative control and an exogenous positive control was used as a calibrator. The RT-qPCR primers sequences are described in ESM Table 6.

### Nonsense-mediated mRNA decay (NMD) cycloheximide inhibition assay and next-generation sequencing

At stage 3 of the differentiation protocol, heterozygous corrected HEL118.3 #*RFX6*^+/−^ cells were treated with different concentrations (25, 50 and 100 µg/ml) of cycloheximide (Sigma-Aldrich, 01810; USA) for durations of 3 and 5 h. Total RNA was collected and reversely transcribed as described above but without using random hexamers. Primers with Illumina sequences attached were designed and used to amplify 120 bp in the variant region of the *RFX6* cDNA, and 10 µl (5 ng/µl) were sequenced using next-generation sequencing (NGS) HiSeq PE100. The percentages of variant and corrected filtered reads from each of the alleles were calculated and plotted for the non-treated control and the cycloheximide treated samples. Primer sequences used for NGS cDNA amplification are described in ESM Table 1.

### Ultra-deep bulk RNA-seq analysis

We performed ultra-deep RNA-seq analysis for H1 *RFX6*^+/+^ and *RFX6*^−/−^ at stage 3 and stage 5, and for *RFX6*^+/+^ and *RFX6*^+/−^ at stage 7 (SC-islets). Following mRNA library preparation using NEBNext Ultra II Directional RNA kit (New England Biolabs, Ipswich, USA), sequencing was performed using NovaSeq SP 2×100 bp v1.5 chemistry (Illumina, San Diego, USA). Preparation of RNA library and transcriptome sequencing was performed by the Sequencing laboratory of Institute for Molecular Medicine Finland FIMM Technology Centre, University of Helsinki. The raw data were filtered with cutadapt [34] to remove adapter sequences (with a minimum overlap of 5 bp), ambiguous (N) and low-quality bases (Phred score <25). We also excluded read pairs that were too short (<25 bp) after trimming. The filtered read pairs were mapped to the human reference genome (GRCh38) with STAR aligner [35]. Gene expression was counted from read pairs mapping to exons using featureCounts in Rsubreads [36], using GENCODE (GRCh38.p13) genome annotations [37]. Duplicate, chimeric and multimapping reads were excluded, as well as reads with low mapping scores (MAPQ <10). We obtained on average 31 million uniquely mapped read pairs per sample (between 27 million and 38 million).

We removed genes with very low or no expression, such as non-polyadenylated genes, from the analysis (<50 reads across all samples). The read count data were analysed with DESeq2 [38], comparing the expression differences between knockout and control samples in the different developmental stages. For stages 3 and 5 we compared the homozygous *RFX6*^−/−^ variant with the control *RFX6*^+/+^ samples. Since *RFX6*^−/−^ cells failed to differentiate into endocrine lineage, we compared the heterozygous *RFX6*^+/−^ (clone 3G) to the control *RFX6*^+/+^ samples at stage 7 week 2. Each genotype per stage had four biological replicates. Genes were considered differentially expressed if the false discovery rate (FDR)-adjusted *p* values were <0.01. Principal component analysis (PCA) was calculated with prcomp (https://www.r-project.org/) using normalised log1p-transformed read counts. The differentially expressed genes (FDR<0.01) were analysed for enrichment separately for the up- and downregulated genes using clusterProfiler [39] against Reactome [40], KEGG [41], Disease ontology [42] and Gene ontology [43] databases. For heatmaps we used log1p-transformed normalised read counts, centred around gene mean using ComplexHeatmap [44]. For the Venn diagram we used ggvenn (https://github.com/yanlinlin82/ggvenn). The rest of the RNA-seq figures were made with ggplot2 [45]. Further details of the tools and software used for RNA-seq analysis are given in ESM Methods. The results from the gene expression analysis together with the raw sequences were deposited to the Gene Expression Omnibus (https://www.ncbi.nlm.nih.gov/geo/), with accession no. GSE234289.

### Static and dynamic GSIS

For static GSIS assay, 50 aggregates were randomly picked and preincubated in 2.8 mmol/l glucose Krebs buffer in a 12-well plate placed on a rotating platform at 95 rev/min for 90 min at 37°C. Aggregates were then washed with Krebs buffer and sequentially incubated in Krebs buffer containing 2.8 mmol/l glucose, 16.8 mmol/l glucose and 2.8 mmol/l glucose plus 30 mmol/l KCl, on the rotating platform for periods of 30 min each. Samples of 200 µl were collected from each treatment and stored at −80°C for insulin ELISA measurements. Dynamic tests of insulin secretion were carried out using a perifusion apparatus (Suprafusion SF-06; Brandel, USA) with a flow rate of 0.25 ml/min and sampling every 4 min. Samples from each fraction collected were analysed using insulin ELISA (Mercodia, Uppsala, Sweden). Following static and dynamic tests of insulin secretion, the SC-islets were collected and the total insulin and DNA contents were analysed. Insulin content results were normalised to the DNA content of the beta cell fraction, calculated from flow cytometry insulin-positive cell percentage.

### Electrophysiology

SC-islets were dispersed into single cells in cell dissociation buffer (Thermo Fisher Scientific) supplemented with trypsin (0.005% wt/vol.; Life Technologies, USA), washed and plated in serum-containing medium on 22 mm polylysine-coated coverslips, allowed to settle overnight, and then transduced with adenovirus coding for enhanced GFP under control of the RIP2 promoter to identify beta cells. Patch-clamp recordings were performed using an EPC-9 patch amplifier with PatchMaster v.2×90 software (HEKA Elektronik, Germany). Electrodes (resistance 2–4 MΩ) were pulled from borosilicate glass capillaries, coated with Sylgard and fire-polished. Cells were superfused with an extracellular solution containing (in mmol/l) 138 NaCl, 5.6 KCl, 1.2 MgCl_2_, 2.6 CaCl_2_, 10 glucose and 5 HEPES, pH 7.4 adjusted with NaOH at a rate of 0.4 ml/min at 32°C. Voltage-dependent currents and exocytosis were measured in whole-cell voltage-clamp mode with an intracellular solution containing (in mmol/l) 125 Cs-glutamate, 10 CsCl, 10 NaCl, 1 MgCl_2_, 0.05 EGTA, 3 Mg-ATP, 0.1 cAMP and 5 HEPES, pH 7.2 adjusted using CsOH. For current-voltage (IV) relationships, the membrane was depolarised from −70 mV to +80 mV (in steps of 10 mV lasting 50 ms each). Currents were compensated for capacitive transients and linear leak by subtracting the sum of the current responses to four depolarisations at 1/4 amplitude (P/4). Na^+^ and Ca^2+^ current components were separated by quantifying the initial peak current (0–5 ms; Na^+^) and average sustained current (5–45 ms; Ca^2+^). Exocytosis was quantified using the lockin module of Patchmaster (30 mV peak-to-peak; 1 kHz); with a train of 14×200 ms depolarisations to 0 mV at 1.4 Hz.

### Exocytosis imaging

To visualise granule exocytosis, cells treated as described for electrophysiology were additionally infected with adNPY-tdOrange2 (a well-established marker for secretory granules) and imaged after 30–36 h using a custom-built lens-type total internal reflection fluorescence (TIRF) microscope based on an AxioObserver Z1 with a ×100/1.45 objective (Zeiss). Excitation was from two diode-pumped solid-state (DPSS) lasers at 491 and 561 nm (Cobolt, Sweden) passed through a cleanup filter (catalogue no. zet405/488/561/×640x; Chroma) and controlled with an acousto-optical tunable filter (AA-Opto). Excitation and emission light were separated using a beamsplitter (catalogue no. ZT405/488/561/640rpc; Chroma). The emission light was separated chromatically onto separate areas of an EMCCD camera (Roper QuantEM 512SC) using an image splitter (Optical Insights) with a cutoff at 565 nm (catalogue no. 565dcxr; Chroma) and emission filters (catalogue nos. ET525/50 m and 600/50 m, Chroma). Scaling was 160 nm per pixel. Cells were imaged in a standard solution containing 138 mmol/l NaCl, 5.6 mmol/l KCl, 1.2 mmol/l MgCl_2_, 2.6 mmol/l CaCl_2_, 10 mmol/l glucose, 5 mmol/l HEPES (pH 7.4 with NaOH), 200 μmol/l diazoxide (to block spontaneous depolarisation; Sigma-Aldrich), and 10 nmol/l exendin-4 (a glucagon-like peptide-1 receptor agonist; Anaspec). Exocytosis was evoked by rapidly depolarising cells with elevated K^+^ (75 mmol/l KCl equimolarly replacing NaCl in the standard solution, by computer-controlled local pressure ejection).

### Cytoplasmic Ca^2+^ imaging

SC-islets were loaded with the fluorescent indicator Fura-2 LR (ion Biosciences) by incubation for 1 h with 1 μmol/l of its acetoxymethyl ester at 37°C in experimental buffer containing in (mmol/l) 138 NaCl, 4.8 KCl, 1.2 MgCl_2_, 2.56 CaCl_2_, 3 glucose and 25 HEPES (pH set to 7.40 with NaOH), and 0.5 mg/ml BSA. After rinsing in indicator-free buffer, the islets were attached to poly-l-lysine-coated coverslips in a 50 μl chamber on the stage of an Eclipse TE2000U microscope (Nikon) and superfused with buffer at a rate of 160 μl/min. The chamber holder and ×40, 1.3-NA objective were maintained at 37°C by custom-built thermostats. An LED light engine (LedHUB, Omicron Laserage Laserprodukte) equipped with 340 and 385 nm diodes and 340/26 nm (centre wavelength/half-bandwidth) and 386/23 nm interference filters (Semrock, IDEX Health & Science, LLC) provided excitation light that was led to the microscope via a liquid light guide. Emission was measured at 510/40 nm using a 400 nm dichroic beamsplitter and an Evolve 512 EMCCD camera (Photometrics). Image pairs at 340/386 nm were acquired every 2 s with the MetaFluor v.7.7 software (Molecular Devices). Cytoplasmic Ca^2+^ concentration ([Ca^2+^]_i_) was calculated from the background-corrected Fura-2 LR 340/380 nm fluorescence excitation ratio from manually defined cell-sized regions of interest. Large numbers of cells (>2000) were analysed at each condition, and differences between experimental groups are therefore unlikely to reflect different proportions of beta and non-beta cells. The data are presented as heatmaps from individual islets and cells and as scatter plots of the absolute [Ca^2+^]_i_ values under different conditions. Calibration of Fura-2LR signal was performed to linearise the ratio signal using solutions without (0 Ca^2+^, 2 mmol/l EGTA) and with saturating Ca^2+^ (10 mmol/l) and 100 µmol/l Fura-2LR salt. The fluorescence ratio was converted to [Ca^2+^]_i_ as described by Grynkiewicz et al [46].

### In vivo animal implantation studies

Animal care and experiments were approved by the National Animal Experiment Board in Finland (ESAVI/14852/2018). NOD.Cg-*Prkdc*^*scid*^* Il2rg*^*tm1Wjl*^/SzJ (NOD-SCID-Gamma [NSG], 005557, Jackson Laboratory; USA, https://www.jax.org/strain/005557) mice were obtained from SCANBUR and housed at Biomedicum Helsinki animal facility, on a 12 h light–dark cycle and fed standard chow ad libitum (2016 Teklad global 16% protein rodent diet, ENVIGO, USA). The temperature was kept at 23°C with 24% relative humidity. Implantations were performed on randomised 4- to 8-month-old male mice. Briefly, stage 6 *RFX6*^−/−^, and stage 7 *RFX6*^+/+^ and *RFX6*^+/−^ SC-islets equivalent to 1.5 million cells (500 clusters) were loaded in PE-50 tubing and implanted under the kidney capsule. Mouse serum samples were collected monthly from the saphenous vein and stored at −80°C for human C-peptide analysis. Blood glucose levels were measured using ContourXT and ContourNext strips. Human-specific C-peptide was measured from plasma samples with the Ultrasensitive C-peptide ELISA kit (Mercodia, Uppsala, Sweden). To test the function of the SC-islet grafts, mice were subjected to an IPGTT. Before the test, mice were fasted 6 h, weighed and blood glucose was measured. Glucose (3 g/kg) was injected intraperitoneally, and blood samples (30 μl) were taken from the saphenous vein after 15, 30, 60 and 90 min to measure blood glucose and circulating human C-peptide levels by ELISA.

### Quantification and statistical analysis

Data were collected from at least three independent differentiation experiments. Blinding was applied for immunohistochemical quantification. Morphological data represent population-wide observation from independent differentiation experiments. All representative images in the figures were reproducible in all the experiments performed and are followed by a quantification panel for all the experiments; the *n* value represents independent differentiation experiments, unless otherwise stated. Bar graphs are represented showing all the individual data points. Statistical methods used are described in each figure legend and individual method section. The results are presented as the mean ± SD unless otherwise stated. *p* values<0.05 were considered statistically significant.

## Results

### Impact of heterozygous and homozygous ***RFX6*** frameshift variant p.His293LeufsTer7 on diabetes development

**Fig. 1:**
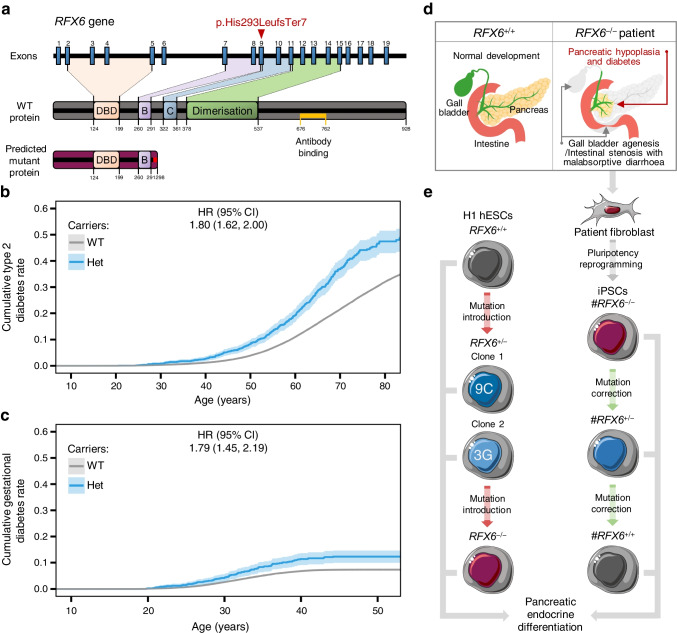
Impact of heterozygous and homozygous *RFX6* frameshift variant p.His293LeufsTer7 on diabetes development. (**a**) The human *RFX6* gene and the encoded WT and predicted mutant proteins. The WT RFX6 is a 928-amino-acid protein containing a DNA binding domain (DBD) and three dimerisation domains (B, C and Dimerisation). The *RFX6* frameshift variant p.His293LeufsTer7 contains the DBD, dimerisation domain B and a frameshift making an early stop codon at 298. The antibody used in this study binds to the amino acid sequence from 676–762 (shown in yellow). (**b**) Survival curve stratified by *RFX6* genotype (WT, *n*=439,416 and Het; heterozygous carriers,* n*=1318) and adjusted HR for type 2 diabetes risk. The mean ± SD age at onset for Het and WT was 57.48±12.87 and 59.91±12.43 years, respectively (*p*=2.5×10^−4^). The total number of individuals in the FinnGen dataset was 440,734, which included 71,728 with type 2 diabetes. (**c**) Survival curve stratified by *RFX6* genotype (WT, *n*=253,803 and Het; heterozygous carriers, *n*=814) and adjusted HR for gestational diabetes risk. The total number of individuals in the FinnGen dataset was 254,617, which included 16,802 with gestational diabetes. (**d**) Schematic showing the patient with Mitchell–Riley syndrome’s clinical manifestations (pancreatic hypoplasia with neonatal diabetes, gall bladder agenesis and intestinal stenosis with malabsorptive diarrhoea). (**e**) Schematic of generating *RFX6* allelic series of the frameshift variant in H1 hESCs and patient-derived iPSCs, followed by directed differentiation into pancreatic endocrine cells. Panel (**e**) was created using Servier Medical Art. Survival plots (**b**, **c**) were generated using survminer and adjusted HRs were calculated using Cox proportional hazards model adjusting for age, sex and principal components PC1–PC10

A two-nucleotide deletion of the *RFX6* gene in exon 9 (c.878_879delAC) leads to a frame shift at codon 293 and an early stop codon at 298 (p.His293LeufsTer7), instead of the 928 amino acid wild-type (WT) protein [47]. While the WT protein contains a winged-helix RFX6 DNA binding domain and three dimerisation domains mediating both hetero- and homodimeric interactions [6], this frameshift variant is predicted to contain only the DNA binding domain and the first dimerisation domain (Fig. 1a). However, in silico protein localisation prediction tools identified the loss of a nuclear localisation sequence, resulting in a predicted cytoplasmic localisation, which is unlikely to interfere with the DNA binding of the WT protein (data not shown) [48–50].

We assessed the impact of this heterozygous frameshift variant on the lifetime risk of type 2 and gestational diabetes in the FinnGen dataset (data freeze R11), containing data from 440,734 individuals. The 1,318 heterozygous carriers of the variant showed 80% higher risk of type 2 diabetes (*p*=6.8×10^−28^), with, on average, a 2 year earlier age of onset (Fig. 1b), and 79% higher risk of gestational diabetes (*p*=2.3×10^−8^) (Fig. 1c). Interestingly, the carriers also demonstrated a reduced BMI (β=−0.12 SD, *p*=1.3×10^−4^ and *n*=321,672) compared with non-carriers.

We identified one male individual carrying this frameshift variant in homozygous form. This boy is currently alive at 10 years of age. He was diagnosed with Mitchell–Riley syndrome at birth, manifesting pancreatic hypoplasia, neonatal diabetes, gall bladder agenesis, intestinal stenosis and malabsorptive diarrhoea (Fig. 1d). The individual had low birth size, which recovered gradually after administering total parenteral nutrition (ESM Fig. 1a). At the age of 4 years, magnetic resonance cholangiopancreatography showed a hypoplastic pancreas of 4 ml (data not shown); the average volume is 20 ml for healthy individuals of the same age [51]. Blood glucose was normal at birth but reached hyperglycaemic levels at the age of 4 days. Insulin treatment was started at a daily dose of 0.1 U/kg, as C-peptide was barely detectable (ESM Fig. 1b). Plasma glucagon was undetectable at birth but, curiously, it normalised gradually over the course of 4 months (ESM Fig. 1c). In contrast, PP dropped below the detection limit (ESM Fig. 1d). Pancreatic exocrine function displayed severe impairment (ESM Fig. 1e). Considering the characteristic clinical features of this homozygous frameshift variant aligning with typical Mitchell–Riley syndrome, and the predicted loss of nuclear localisation of the protein, we have denoted this variant as a loss-of-function variant *RFX6*^−/−^.

To study the p.His293LeufsTer7 variant in more detail, we generated two isogenic allelic series stem cell models. In the first model, we introduced the variant in H1 hESCs. We validated two heterozygous clones (*RFX6*^+/−^ 9C and *RFX6*^+/−^ 3G) and one homozygous (*RFX6*^−/−^) clone (Fig. 1e and ESM Fig. 2). In the second model, we generated patient-derived iPSCs from the homozygous patient and then corrected the variant heterozygously and homozygously, creating the iPSC lines #*RFX6*^−/−^, *#RFX6*^+/−^ and *#RFX6*^+/+^ (Fig. 1e and ESM Fig. 3).

### RFX6 controls the transcriptional network of pancreatic development

**Fig. 2:**
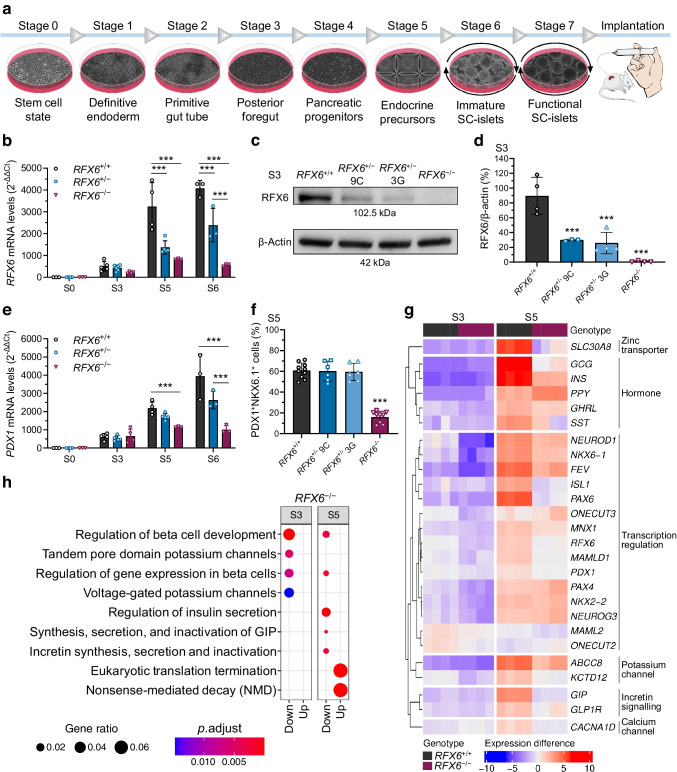
RFX6 controls the transcriptional network of pancreatic development. (**a**) Schematic of H1 SC-islet differentiation protocol. Stages 1–4 in monolayer, stage 5 in microwells and stages 6–7 in suspension culture, followed by implantation under kidney capsule of NSG mice. (**b**) Relative gene expression levels of *RFX6* at stage 0 (S0), S3, S5 and S6 (*n*=3–5). (**c**) Protein immunoblots of RFX6 and β-actin for the isogenic H1 clones (9C and 3G) at S3. (**d**) Percentage of RFX6 protein band densitometry normalised to β-actin bands, quantified from (**c**) (*n*=3 or 4). (**e**) Relative gene expression levels of *PDX1* at S0, S3, S5 and S6 (*n*=3–5). (**f**) Percentage of PDX1^+^ and NKX6.1^+^ cells at S5 measured by flow cytometry (*n*=6–10). (**g**) Heatmap showing the relative differences in gene expression of *RFX6*^*+/+*^ and *RFX6*^−*/*−^ at S3 and S5 (*n*=4). Using log1p transformed normalised read count, with sample specific expression subtracted from gene averages. Each gene is differentially expressed in one or both of the stages, FDR<0.01. (**h**) Enrichment analysis of the differentially expressed genes between *RFX6*^*+/+*^ and *RFX6*^−*/*−^ at S3 and S5. The upregulated and downregulated genes (FDR<0.01) were analysed for enrichment against the Reactome database separately for S3 and S5 comparisons. Statistical significance was measured using two-way ANOVA with Tukey’s test for multiple comparisons correction in (**b**, **e**), and one-way ANOVA with Tukey’s test for multiple comparisons correction in (**d**, **f**). Data are presented as means ± SD; ***p<0.001

We differentiated the generated hESCs towards functional SC-islets using our optimised seven-stage protocol [28, 29] (Fig. 2a). Since *RFX6* expression started to increase at the posterior foregut stage (S3) (Fig. 2b), the frameshift variant did not affect the earlier stage of definitive endoderm induction (data not shown). However, levels of *RFX6* gene expression in both heterozygous *RFX6*^+/−^ and homozygous *RFX6*^−/−^ cells were reduced (Fig. 2b). The loss of RFX6 in *RFX6*^−/−^ cells was confirmed by immunoblotting at S3 and, interestingly, both heterozygous clones showed significant reduction in RFX6 protein levels at this stage, indicating haploinsufficiency (Fig. 2c,d). The lack of RFX6 in *RFX6*^−/−^ cells was associated with a significant reduction in expression of *PDX1*, a key transcription factor in pancreatic development (Fig. 2e). Subsequently, the percentage of PDX1^+^ NKX6.1^+^ and the emergence of cells positive for chromogranin A (CHGA) were significantly reduced (markers of pancreatic endocrine lineages), while both heterozygous clones were similar to *RFX6*^+/+^ (Fig. 2f and ESM Fig. 4).

Since the quantity and quality of pancreatic progenitors and endocrine precursors were impaired in *RFX6*^−/−^, but not in *RFX6*^+/−^ cells, we ran bulk RNA-seq analysis to identify differentially expressed genes between *RFX6*^−/−^ and *RFX6*^+/+^ at stages 3 and 5 (S3 and S5; ESM Fig. 5). In *RFX6*^−/−^ cells, the transcription factor regulatory network orchestrating the development of the pancreatic lineage was downregulated at S3 including *RFX6*, *PDX1*, *NKX6.1* (also known as *NKX6-1*), *FEV*, *PAX4*, *NEUROG3* and *NEUROD1* (Fig. 2g). While most of these genes remained downregulated at S5 in addition to *ISL1* and *PAX6*, expression levels of *NEUROG3* and *NKX2.2* recovered, with a significant increase in *PAX4* compared with *RFX6*^+/+^. Interestingly, expression levels of all pancreatic hormones were downregulated at S5, except for *PPY*, which was significantly upregulated (Fig. 2g and ESM Fig. 6a). In agreement with these findings, Reactome enrichment analysis showed downregulation of pathways involved in beta cell development and gene expression in *RFX6*^−/−^ cells. Additionally, voltage-gated potassium channels were reduced at S3, and the regulation of insulin secretion, and glucose-dependent insulinotropic polypeptide (GIP) synthesis and secretion were reduced at S5 (Fig. 2h).

We further confirmed these findings in our isogenic iPSC model using a modified version of a simplified monolayer differentiation protocol [31]. As observed with hESCs, the emergence of cells positive for pancreatic and duodenal homeobox 1 (PDX1) in #*RFX6*^−/−^ was not compromised, while *RFX6* and *PDX1* gene expression and the generation of NK6 homeobox 1 (NKX6.1)-positive pancreatic progenitors were significantly reduced (ESM Fig. 7 and ESM Fig. 8a, b). We then investigated if the cells carrying the frameshift variant exhibit reduced *RFX6* expression due to NMD. We treated #*RFX6*^+/−^ cells with the NMD inhibitor cycloheximide at S3 and quantified the reads of variant and corrected transcripts. In the non-treated samples, the percentage of frameshift variant cDNA was only 15%, while treatment with cycloheximide gradually normalised the expression to ~48% in a dose-dependent manner, confirming that the frameshift variant mRNA was subjected to NMD (ESM Fig. 8c). At the endocrine stage (S6), #*RFX6*^−/−^ cells lacked the expression of the five pancreatic hormones, insulin, glucagon, somatostatin, PP and ghrelin, while this phenotype was rescued in both the heterozygous and homozygous corrected cells (ESM Fig. 8d, e).

### Persistent expression of SRY-box transcription factor 9 and neurogenin 3 in homozygous ***RFX6***^−/−^ cells

**Fig. 3:**
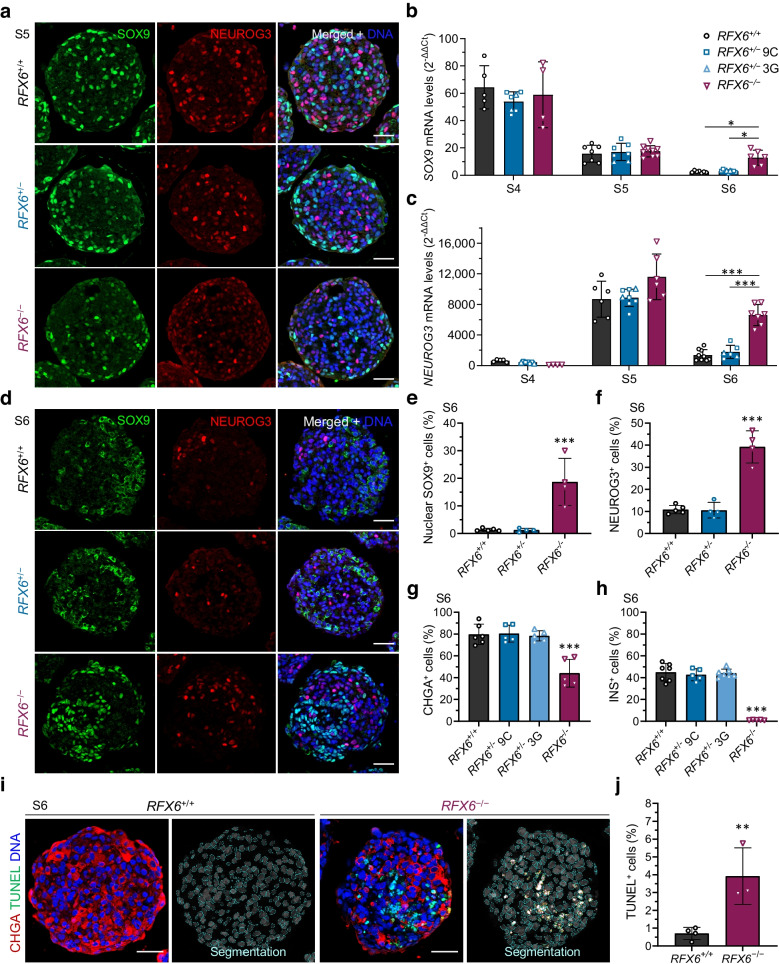
Persistent expression of SOX9 and NEUROG3 in homozygous *RFX6*^−*/*−^ cells and increased apoptosis. (**a**) Immunohistochemistry showing SOX9^+^ and NEUROG3^+^ cells at stage 5 (S5). Scale bar, 50 µm. (**b**, **c**) Relative gene expression levels of *SOX9* (**b**) and *NEUROG3* (**c**), comparing all the cell lines at S4, S5 and S6 (*n*=4–9). (**d**) Immunohistochemistry for SOX9^+^ and NEUROG3^+^ cells at S6. Scale bar, 50 µm. (**e**, **f**) Percentages of SOX9^+^ (**e**) and NEUROG3^+^ cells (**f**) at S6 quantified from (**d**) (*n*=4–5). (**g**, **h**) Percentage of CHGA^+^ (**g**) and insulin (INS)^+^ cells (**h**) at S6 quantified by flow cytometry (*n*=5–7). (**i**) Immunohistochemistry showing TUNEL^+^ and CHGA^+^ cells for the *RFX6*^*+/+*^ and *RFX6*^−*/*−^ clones at S6. Scale bar, 50 µm. (**j**) Percentage of TUNEL^+^ cells at S6 quantified from (**i**) (*n*=3–4). Statistical significance was measured using two-way ANOVA with Tukey’s test for multiple comparisons correction in (**b**, **c**), one-way ANOVA with Tukey’s test for multiple comparisons correction in (**e**–**h**) and two-tailed unpaired *t* test in (**j**). Data are presented as means ± SD; *p<0.05, **p<0.01, ***p<0.001

We further studied the endocrine precursors in the 3D model of hESCs and found a peculiar dysregulation of SRY-box transcription factor 9 (SOX9) and neurogenin 3 (NEUROG3). Normally, when SOX9 is highly expressed in the pancreatic epithelium it induces the proendocrine transcription factor NEUROG3, which upon reduction of notch signalling promotes the endocrine lineage differentiation program [52]. Indeed, in our S4 pancreatic progenitors, *SOX9* was highly expressed across all lines (Fig. 3b and ESM Fig. 4a). Upregulation of NEUROG3 expression at S5 was associated with downregulation of *SOX9* expression (Fig. 3a–c). A further decrease in *SOX9* expression was noticed in the immature S6 SC-islets in *RFX6*^+/+^ and *RFX6*^+/−^ but not in *RFX6*^−/−^ cells (Fig. 3b). Interestingly, *NEUROG3* expression increased at S5 in *RFX6*^−/−^ cells, similar to the observation in bulk RNA-seq, but the difference did not reach statistical significance. However, it was significantly higher than in the other genotypes at S6 (Fig. 3c). The pattern of SOX9 and NEUROG3 expression was similar between the genotypes in S5 endocrine precursors, as shown by immunohistochemistry (Fig. 3a). However, the pattern was completely altered at S6; whereas SOX9 was detected mainly in the cytoplasm of *RFX6*^+/+^ and *RFX6*^+/−^ cells, nuclear SOX9 expression persisted in *RFX6*^−/−^ cells (Fig. 3d). The numbers of nuclear SOX9^+^ cells and NEUROG3^+^ cells were significantly increased in *RFX6*^−/−^ cells (Fig. 3e,f). Since NEUROG3 expression induces the endocrine lineage specification, we investigated the expression of the endocrine marker CHGA. While ~80% of the cells were CHGA^+^ in *RFX6*^+/+^ and *RFX6*^+/−^ cells, only ~43% were CHGA^+^ in *RFX6*^−/−^ cells, with less than 1% being insulin-positive (Fig. 3g,h and ESM Fig. 9a). Most CHGA^+^ cells in *RFX6*^−/−^ cells displayed positivity for solute carrier family 18 member A1 (SLC18A1), a marker of enterochromaffin cells (a subpopulation commonly observed in various differentiation protocols, including ours [28, 53, 54]), although they were disproportionately increased in *RFX6*^−/−^ (ESM Fig. 9b). A significant increase in apoptosis was detected in *RFX6*^−/−^ cells by TUNEL assay at the end of S6 (Fig. 3i,j), and the cells did not survive for more than 2 days at the final maturation stage 7 (S7). This suggests that the absence of RFX6, and consequently the failure to specify pancreatic endocrine precursors, results in progressive cell death.

### ***RFX6*** haploinsufficiency impairs insulin secretion of beta cells

Quantifying all endocrine populations (positive for insulin, glucagon, somatostatin, PP, ghrelin and SLC18A1), we verified an extremely limited presence of pancreatic hormone cells in *RFX6*^−/−^ cells, with no significant difference between *RFX6*^+/+^ and *RFX6*^+/−^ cells. Although there was a slight increase in enterochromaffin cells (SLC18A1^+^) in *RFX6*^+/−^, the difference did not reach statistical significance (ESM Fig. 10).

**Fig. 4:**
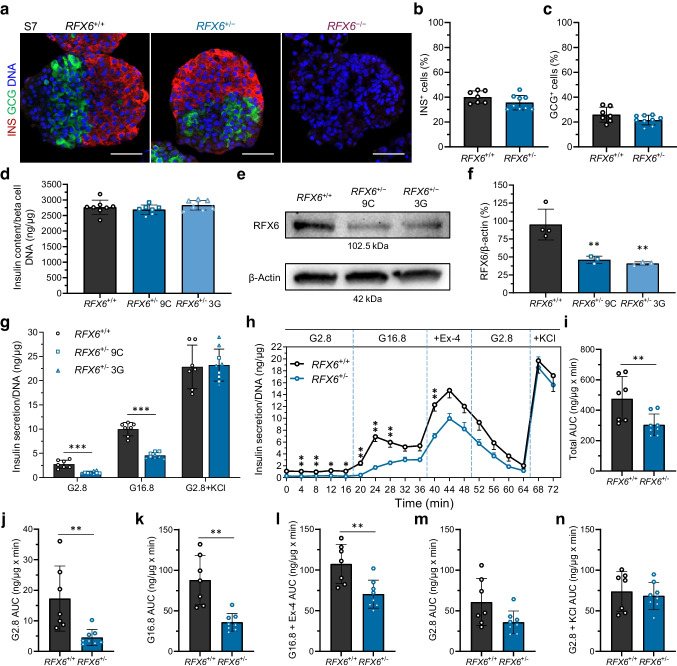
*RFX6* haploinsufficiency impairs insulin secretion of beta cells. (**a**) Immunohistochemistry showing insulin-positive (INS^+^) and glucagon-positive (GCG^+^) cells for *RFX6*^+/+^ and *RFX6*^+/−^ SC-islets at stage 7 week 2 (S7w2), and for *RFX6*^−/−^ at stage 7 day 2 (S7d2). Scale bar, 50 µm. (**b**, **c**) Percentage of INS^+^ (**b**) and GCG^+^ cells (**c**) for *RFX6*^+/+^ and *RFX6*^+/−^ SC-islets at S7w2 measured by flow cytometry (*n*=7–9). (**d**) Insulin content of *RFX6*^+/+^ and *RFX6*^+/−^ SC-islets at stage 7 week 3 (S7w3) normalised to the DNA content of beta cells (*n*=7 or 8). (**e**) Protein immunoblots of RFX6 and β-actin for *RFX6*^+/+^ and *RFX6*^+/−^ SC-islets at S7w2. (**f**) Percentage of RFX6 protein bands densitometry normalised to β-actin bands, quantified from (**e**) (*n*=3 or 4). (**g**) Static insulin secretion at S7w3 at low 2.8 mmol/l glucose (G2.8), followed by high 16.8 mmol/l glucose (G16.8) and then depolarisation with G2.8+30 mmol/l KCl, normalised to the DNA content of the SC-islets (*n*=7–9). (**h**) Dynamic insulin secretion responses to perifusion (4 min intervals) with G2.8, G16.8, G16.8+12 nmol/l exendin-4 (Ex-4) and G2.8+30 mmol/l KCl, normalised to the DNA content of the SC-islets (*n*=7 or 8). (**i**) Total AUC, quantified from (**h**) (*n*=7 or 8). (**j**–**n**) AUC for individual phases, quantified from (**h**) (*n*=7 or 8). Statistical significance was measured using unpaired *t* test in (**b**, **c**, **i**–**n**), using one-way ANOVA with Tukey’s test for multiple comparisons correction in (**d**, **f**) and using two-tailed unpaired multiple *t* tests in (**g**, **h**). Data are presented as means ± SD; *p<0.05, **p<0.01, ***p<0.001

Since *RFX6*^−/−^ cells failed to survive at S7, we continued to study the function of only the WT and heterozygous cells. The proportions of insulin- and glucagon-positive cells (markers of beta cells and alpha cells, respectively) and total insulin content were similar between the two genotypes (Fig. 4a–d and ESM Fig. 11a). We confirmed the persistent *RFX6* haploinsufficiency at this stage using protein immunoblotting, showing 54.2% reduction in protein expression (Fig. 4e,f). GSIS in a static assay, normalised to DNA content, showed decreased insulin secretion in *RFX6*^+/−^ compared with WT cells in both basal and high glucose conditions (62% and 54% reduction, respectively), while KCl-stimulated insulin secretion showed no difference (Fig. 4g). However, glucose stimulatory index was similar between *RFX6*^+/+^ and *RFX6*^+/−^ cells (ESM Fig. 11b). Correspondingly, dynamic perifusion assays confirmed the reduced insulin secretion in both glucose conditions (Fig. 4h). They also revealed a blunted first-phase response, as well as lower insulin secretion when stimulated with the glucagon-like peptide 1 (GLP-1) analogue exendin-4. Insulin secretion shut down effectively when the glucose concentration was decreased back to 2.8 mmol/l in both genotypes, followed by a normal maximum secretion capacity with KCl depolarisation (Fig. 4h). Total AUC for the entire perifusion assay was significantly reduced in *RFX6*^+/−^ cells, including the individual phases of low glucose, high glucose, and high glucose with exendin-4 (Fig. 4i–n). These findings highlight that lower RFX6 expression in the heterozygous cells reduces the insulin secretion capacity in basal and stimulatory glucose conditions without impacting beta cell number or insulin content.

### Reduced [Ca^2+^]_i_ in basal and depolarised conditions in ***RFX6***^+/−^ SC-islets

**Fig. 5:**
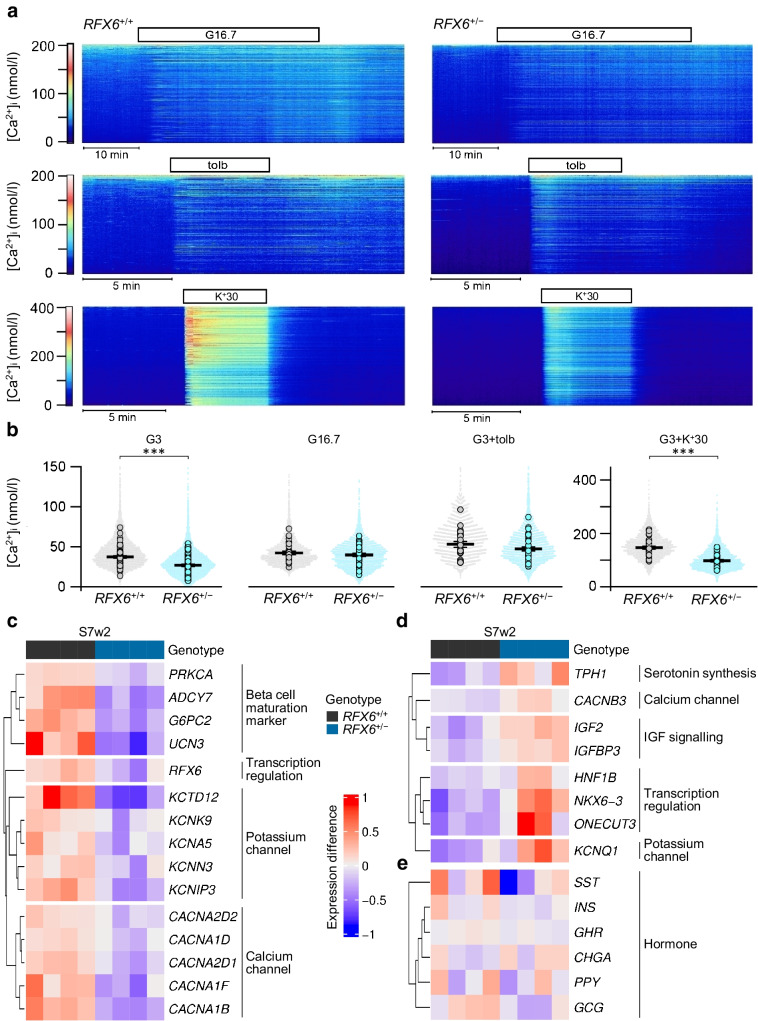
Reduced [Ca^2+^]_i_ in basal and depolarised conditions in *RFX6*^+/−^ SC-islets. (**a**) Heatmaps of [Ca^2+^]_i_ recorded with Fura-2 LR in cells within SC-islets under basal conditions of 3 mmol/l glucose (G3) and after stimulation with 16.7 mmol/l glucose (G16.7), G3+1 mmol/l tolbutamide (tolb) and G3+30 mmol/l K^+^ (K^+^30). Each heatmap shows the responses of all cells in one islet. (**b**) Quantification of [Ca^2+^]_i_ responses from experiments as in (**a**). Time-averaged [Ca^2+^]_i_ for all individual cells (dots) and average values for all cells in each islet (circles). The bars represent means ± SEM for the averaged islet responses. *n*=no. of islets (total cell number in parenthesis): G3 WT, *n*=65 (6933); G3 *RFX6*^+/−^, *n*=75 (8502); G16.7 WT, *n*=29 (3203); G16.7 *RFX6*^+/−^, *n*=42 (4853); tolb WT, *n*=23 (2720); tolb *RFX6*^+/−^, *n*=39 (4692); K^+^30 WT *n*=32 (3639); and K^+^30 *RFX6*^+/−^, *n*=37 (4320). Statistical analyses with Student’s unpaired two-tailed *t* test, ***p<0.001. (**c**) Heatmap of downregulated genes in *RFX6*^+/−^ SC-islets at stage 7 week 2 (S7w2). Statistically significant genes (*ADCY7*, *G6PC2*, *UCN3*, *KCTD12*, *KCNIP3* and *CACNA1B*), FDR<0.01 (*n*=4). (**d**) Heatmap of upregulated genes in *RFX6*^+/−^ SC-islets at S7w2. All genes except *IGFBP3* are statistically significant, FDR<0.01 (*n*=4). (**e**) Heatmap of hormone gene expression in *RFX6*^+/+^ and *RFX6*^+/−^ SC-islets at S7w2. None of the genes show statistically significant differences (*n*=4). Heatmaps (**c**–**e**) show log1p transformed normalised read count, with sample-specific expression subtracted from gene averages

To mechanistically understand the reduced glucose sensitivity of *RFX6*^+/−^ SC-islets, we explored the stimulus–secretion coupling machinery of SC-islet beta cells by analysing [Ca^2+^]_i_, ion channel conductance and exocytosis. The [Ca^2+^]_i_ was recorded from individual cells in SC-islets loaded with Fura-2LR. In line with reduced basal insulin secretion, [Ca^2+^]_i_ at 3 mmol/l glucose was significantly lower in *RFX6*^+/−^ than in *RFX6*^+/+^ SC-islets (Fig. 5a,b). Most of the SC-islet cells responded to 16.7 mmol/l glucose with [Ca^2+^]_i_ elevation, indicating beta cell identity. The glucose-induced [Ca^2+^]_i_ increase was moderately reduced in *RFX6*^+/−^ SC-islets but the difference did not reach statistical significance. Blockade of K_ATP_ channels with 1 mmol/l tolbutamide increased [Ca^2+^]_i_ to an extent comparable with glucose stimulation. Depolarisation with 30 mmol/l K^+^ resulted in a more pronounced [Ca^2+^]_i_ increase, which was significantly reduced in *RFX6*^+/−^ compared with *RFX6*^+/+^ SC-islets (Fig. 5a,b). Patch-clamp recordings showed no difference in amplitude or voltage-dependence of total and nifedipine-sensitive Ca^2+^ currents or of Na^+^ currents (ESM Fig. 11c, d). Exocytosis was measured in single cells as changes in membrane capacitance. The pre-stimulatory capacitance was similar in control and *RFX6*^+/−^ cells, indicating no difference in cell size (ESM Fig. 11e). A train of depolarisations (14×200 ms) resulted in slightly larger capacitance increase in *RFX6*^+/−^ cells (ESM Fig. 11f, g) but the difference was not statistically significant. Exocytosis was also measured by TIRF microscopy in cells expressing the granule marker NPY-tdmOrange2. Control and *RFX6*^+/−^ cells showed similar granule density at the plasma membrane and comparable degree of granule fusion events following depolarisation with high K^+^ combined with diazoxide (ESM Fig. 11h, i). Together, these experiments indicate that *RFX6*^+/−^ SC-islets have no general impairment of exocytosis capacity and that the reduced GSIS is most likely explained by other factors, such as altered generation of metabolic coupling factors that amplify Ca^2+^-triggered exocytosis.

To further investigate the findings of lower insulin secretion and reduced [Ca^2+^]_i_, we performed transcriptome analysis at S7 using bulk RNA-seq (ESM Fig. 5). The gene expression levels of *RFX6* were downregulated in the heterozygous cells, in line with the observed lower protein expression and functional haploinsufficiency. Concomitantly, beta cell maturation markers, such as *G6PC2* [28], *UCN3* [55], *ADCY7* [56] and *PRKCA* [57–59], together with voltage-gated calcium and potassium channels, were reduced compared with expression in *RFX6*^+/+^ cells (Fig. 5c and ESM Fig. 12a)*.* In contrast, pancreatic progenitor genes such as, *ONECUT3* [60], *NKX6-3 *[61, 62] and *HNF1B* [63] were upregulated (Fig. 5d and ESM Fig. 12b), indicating an immature phenotype of the heterozygous SC-islets. Notably, the serotonin biosynthesis gene *TPH1* was significantly increased, aligning with the observed, albeit non-significant, rise in enterochromaffin cells (ESM Fig. 10e), and consistent with findings in *Rfx6*-deficient mice [64]. Moreover, upregulation of the disallowed genes *IGF2* [65], *CACNB3* [66, 67] and *KCNQ1* [68, 69] was detected (Fig. 5d and ESM Fig. 12b). However, the gene expression of pancreatic hormones was unaltered (Fig. 5e and ESM Fig. 12c). These findings illustrate the immature phenotype of *RFX6*^+/−^ SC-islets, with reduced [Ca^2+^]_i_ and insulin secretion.

### Heterozygous ***RFX6*** frameshift variant beta cells present lower insulin secretion in vivo

**Fig. 6:**
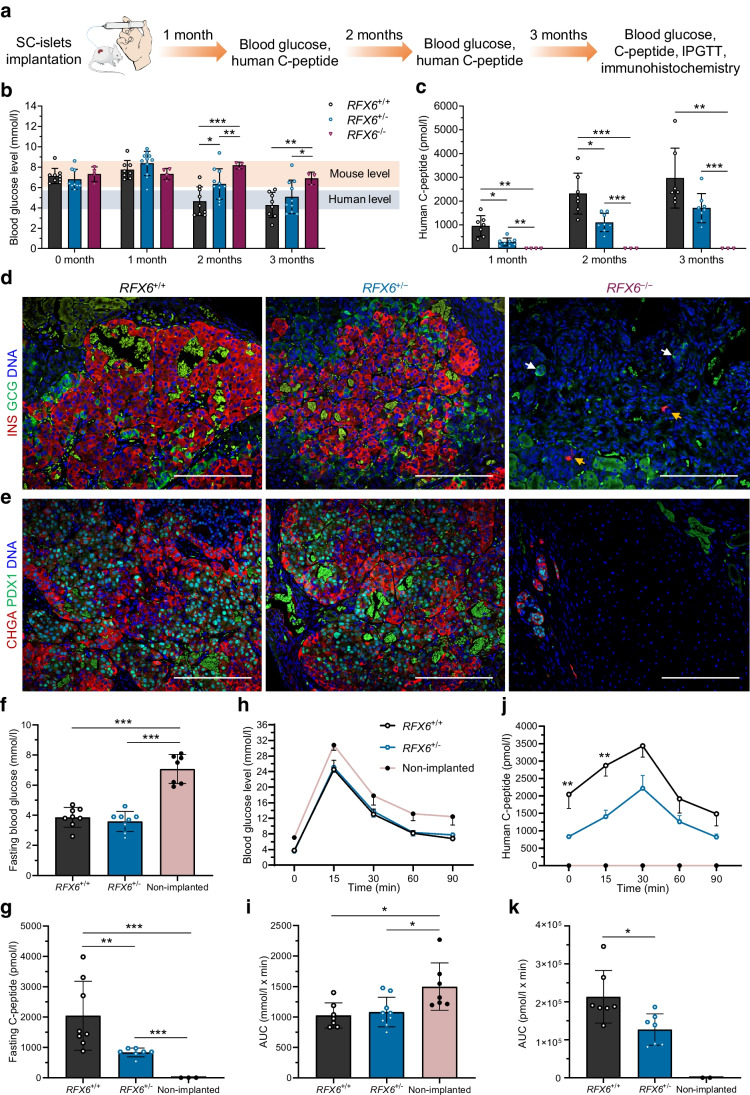
Heterozygous *RFX6*^+/−^ beta cells present lower insulin secretion in vivo. (**a**) Schematic showing the data points and sampling post implantation of SC-islets under the kidney capsule of NSG mice. (**b**) Random blood glucose levels measured monthly post implantation (*n*=3–11). (**c**) Random human C-peptide levels measured monthly post implantation (*n*=3–8). (**d**) Immunohistochemistry showing insulin-positive (INS^+^) and glucagon-positive (GCG^+^) cells at month 3 post implantation. In *RFX6*^−/−^ cells, yellow arrows point to INS^+^ and white arrows point to GCG^+^. Scale bar, 200 µm. (**e**) Immunohistochemistry showing CHGA^+^ and PDX1^+^ cells at month 3 post implantation. Scale bar, 200 µm. (**f**) Fasting blood glucose levels measured at month 3 post implantation (*n*=7 or 8). (**g**) Fasting human C-peptide levels measured at month 3 post implantation (*n*=3–8). (**h**) Blood glucose levels during an IPGTT of fasted mice at month 3 post implantation (*n*=7–9). (**i**) AUC quantified from (**h**) (*n*=7–9). (**j**) Human C-peptide during an IPGTT of fasted mice at month 3 post implantation (*n*=2–7). (**k**) AUC quantified from (**j**) (*n*=2–7). Statistical significance was measured using two-way ANOVA with Tukey’s test for multiple comparisons correction in (**b**, **c**), one-way ANOVA with Tukey’s test for multiple comparisons correction in (**f**–**i**) and two-tailed unpaired *t* test between *RFX6*^+/+^ and *RFX6*^+/−^ in (**j**, **k**). Data are presented as means ± SD, except for (**h**, **j**) showing mean ± SEM; *p<0.05, **p<0.01, ***p<0.001

To investigate whether the reduced insulin response persisted after further maturation of the SC-islets in vivo, we implanted 500 S7 *RFX6*^+/+^ or *RFX6*^+/−^ SC-islets under the kidney capsule of immunocompromised non-diabetic NSG mice. Additionally, we implanted S6 *RFX6*^−/−^ SC-islets to evaluate the fate of the surviving cells in vivo, since they failed to survive in vitro. Blood glucose and human C-peptide levels were measured monthly for 3 months, followed by an IPGTT (Fig. 6a). The normal range of blood glucose levels in random-fed NSG mice is between 6 and 8.5 mmol/l; however, starting from the second month post implantation, the blood glucose levels of the mice implanted with *RFX6*^+/+^ SC-islets dropped to the human level of ~4.6 mmol/l (Fig. 6b). In contrast, mice implanted with *RFX6*^+/−^ SC-islets required an additional month to display human blood glucose levels (Fig. 6b). *RFX6*^−/−^ cells failed to reduce the mouse blood glucose levels. While human C-peptide levels were undetectable in mice implanted with *RFX6*^−/−^ SC-islets, they increased steadily in the mice implanted with *RFX6*^+/+^ and *RFX6*^+/−^ SC-islets. However, throughout the 3 months, the C-peptide levels in mice implanted with *RFX6*^+/−^ SC-islets were approximately half those in their *RFX6*^+/+^-implanted counterparts (Fig. 6c).

Immunohistochemical analysis after 3 months showed abundant insulin-positive and glucagon-positive cells in *RFX6*^+/+^ and *RFX6*^+/−^ grafts, while these cell types were rarely found in *RFX6*^−/−^ implants (Fig. 6d). Similarly, most of the implanted *RFX6*^+/+^ and *RFX6*^+/−^ cells were CHGA-positive, with an abundance of PDX1-positive cells, representing insulin-positive cells (Fig. 6e). In contrast, most cells were negative for both markers in *RFX6*^−/−^ implants and the morphology of cells was distinctly different. To evaluate the origin of these cells, we performed immunohistochemical analysis using a human-specific mitochondrial antibody (hMITO), together with SOX9 immunostaining, that persists in *RFX6*^−/−^ in vitro. Indeed, insulin-positive cells from the *RFX6*^+/+^ implants were positive for hMITO, while the surrounding cells from the mouse tissue were negative (ESM Fig. 13a). The morphologically different cells in the *RFX6*^−/−^ implants were positive for hMITO and nuclear SOX9, compared with scarce cytoplasmic SOX9-positive cells in *RFX6*^+/+^ implants. Both *RFX6*^+/+^ and *RFX6*^−/−^ cells were negative for NEUROG3 expression at 3 months post implantation (ESM Fig. 13b). Since PP cells were found in *Rfx6*^−/−^ mice, we stained for PP and did not detect any PP-positive cells in the *RFX6*^−/−^ grafts (ESM Fig. 13c). This suggests that RFX6 is essential for the development of all pancreatic endocrine cells in humans, including PP cells.

At 3 months post implantation, *RFX6*^+/+^ and *RFX6*^+/−^ implanted mice had a blood glucose level of ~3.9 mmol/l, similar to the fasting level in humans, while non-implanted mice presented the normal mouse blood glucose levels of ~7 mmol/l (Fig. 6f). However, human C-peptide levels in fasting mice implanted with *RFX6*^+/−^ SC-islets were ~60% lower (Fig. 6g). In the IPGTT, both *RFX6*^+/+^ and *RFX6*^+/−^ SC-islets were able to control the abrupt increase in blood glucose levels similarly, and more efficiently, compared with the non-implanted mice, which had higher blood glucose levels at each of the measured time points (Fig. 6h) and in the measured AUC (Fig. 6i). Although the secreted human C-peptide levels of both genotypes had a similar pattern and peaked at 30 min post injection, the levels were lower in the *RFX6*^+/−^ implanted mice (Fig. 6j) and the total secretion AUC was significantly reduced (Fig. 6k). These findings indicate that *RFX6* haploinsufficiency due to the lower gene dose in *RFX6*^+/−^ SC-islets leads to persistent reduction in insulin secretion.

## Discussion

A hierarchy of gene regulatory networks control the development and function of beta cells. Single pathogenic variants in more than 20 transcription factors of this network can lead to diabetes. Importantly, species differences in the developmental and functional processes of beta cells impede their precise study with mouse models. Here, we sought to overcome this limitation using genetically engineered human SC-islets, to investigate the developmental and functional defects of beta cells upon *RFX6* perturbation.

Our findings demonstrate that *RFX6* p.His293LeufsTer7 is a loss-of-function variant with a transcript subjected to nonsense-mediated decay. We confirmed that the lower *RFX6* gene dose in heterozygous *RFX6* SC-islets led to *RFX6* haploinsufficiency, which did not compromise the differentiation capacity of the cells in terms of beta cell number or insulin content, but impaired beta cell function. There is no association between *Rfx6* haploinsufficiency and diabetes in mice, indicating that this phenotype cannot be faithfully modelled in rodents [6]. Similar discrepancies between diabetes development in humans and mice have been reported with variants in *HNF1A*, *HNF1B* and *PAX4*. While pathogenic heterozygous variants in all of these genes cause MODY in humans, heterozygous null mice lack any diabetes phenotype [70–72]. However, our findings are in line with the beta cell-specific *Rfx6* knockout in adult mice [12] and confirm the reported reduction of insulin secretion in islets from donors with type 2 diabetes as a result of reduced *RFX6* levels [14]. Reduced RFX6 expression in *RFX6*^*+/*−^ SC-islets led to decreased levels of the beta cell marker *UCN3* and VDCC expression, lower [Ca^2+^]_i_ and reduced insulin secretion in basal and high glucose levels. Moreover, *RFX6*^*+/*−^ SC-islets showed upregulation of disallowed genes that are reported to negatively impact insulin secretion, such as *IGF2* [65], *CACNB3* [66, 67] and *KCNQ1* [68, 69].

Insulin content of the heterozygous cells was not affected, suggesting that the difference in insulin secretion more likely reflects impaired stimulus–secretion coupling or granule trafficking. However, exocytosis measured either as changes in capacitance or by TIRF imaging of granules at the plasma membrane was not reduced in response to depolarising stimuli. This is in line with intact K^+^-induced insulin secretion in static and dynamic perifusion experiments. Despite slightly reduced gene expression levels of some voltage-gated Ca^2+^ channel components, there was no difference in Ca^2+^ currents, unlike the reduced Ca^2+^-channel activity that was reported in *RFX6* knockdown in the EndoC-βH2 cell line [13]. The reduced basal [Ca^2+^]_i_ and the lower [Ca^2+^]_i_ after membrane depolarisation may instead reflect differences in Ca^2+^ transport or buffering. The lower basal [Ca^2+^]_i_ is consistent with reduced insulin secretion at low glucose. Since the substimulatory [Ca^2+^]_i_ influences granule priming [73], reduced resting [Ca^2+^]_i_ may indirectly impair secretion also at elevated glucose. However, the intact K^+^-induced insulin secretion, and the reduced GSIS despite insignificantly altered [Ca^2+^]_i_, more likely point towards a role for *RFX6* in controlling the metabolic amplifying pathway. The [Ca^2+^]_i_ elevation triggered by K^+^ depolarisation was significantly reduced in *RFX6*^*+/*−^ cells, yet Ca^2+^ influx near insulin granules might still reach a level sufficient to induce a maximum insulin secretion response, as evidenced in both static and dynamic insulin secretion assays. Based on these observations, we suggest insulin secretagogues and/or GLP-1 receptor agonists as treatment to enhance the secretory capacity of beta cells in heterozygous *RFX6* variant carriers.

The heterozygous *RFX6* SC-islets sustained lower insulin secretion in vivo, resulting in delayed lowering of blood glucose post implantation. All of our findings from the heterozygous cell model are consistent with the increased risk of variant carriers to develop diabetes, as shown for both gestational and type 2 diabetes, and previously for MODY with reduced penetrance [15]. Further exploration of the clinical phenotype in variant carriers is warranted. However, two small studies have indicated elevated fasting glucose [15] or 2 h glucose [47] during an OGTT in carriers compared with non-carriers. Additionally, lower fasting and stimulated GIP levels were observed [15]. Consistent with an impact on insulin secretion, carriers displayed a younger age at diagnosis of diabetes and a lower BMI.

The complete loss of RFX6 led to reduced expression of *PDX1*, *NKX6.1*, *FEV*, *ISL1, NEUROD1* and *PAX6* with gradual loss of pancreatic progenitor identity. These genes are in line with some of the reported targets of ChIP-seq analysis of whole pancreatic tissue from adult mice (e.g. *Pdx1*, *Neurod1* and *Nkx6.1*) [74]. Although, the pancreatic markers *NEUROG3*, *NKX2.2* and *PAX4* were reduced at an earlier stage of the differentiation, they recovered at the endocrine precursor stage. This phenotype is similar to that reported in *Rfx6* knockout mice [6, 12], with a difference of reduced *NKX6.1* expression in our model. While gene expression levels of *PPY* were increased in *RFX6*^−/−^ endocrine precursors, PP cells formed in extremely limited numbers in vitro and were not found after 3 months following implantation in vivo, recapitulating the loss of pancreatic polypeptide in the plasma of the Mitchell–Riley syndrome patient, in contrast to the increased numbers of PP cells in the *Rfx6*^−/−^ mouse model [6]. We demonstrate persistent expression of NEUROG3 and SOX9 at the pancreatic endocrine stage in *RFX6*^−/−^ cells, suggesting a negative regulatory role of RFX6 on SOX9 and NEUROG3 expression. Most of the emerging endocrine cells exhibited enterochromaffin cell identity with a pancreatic endocrine specification barricade, followed by increased cell death upon further differentiation in vitro. Reduced generation of the pancreatic progenitor pool and increased apoptosis phenotype could explain the pancreatic hypoplasia seen in homozygous individuals. Implantation of the homozygous cells confirmed persistent SOX9 expression in the majority of the grafted cells, while CHGA-expressing cells were extremely rare. This suggests that RFX6 functions to limit SOX9 expression to allow full endocrine differentiation. Human C-peptide was not detected in mice implanted with *RFX6*^−/−^ cells. The presence of small numbers of insulin-positive and glucagon-positive cells may explain the very low C-peptide levels observed in the patient but not the normal glucagon levels. These normal glucagon levels might be explained by potential extrapancreatic secretion of the hormone [75].

In summary, we highlight the critical role of RFX6 in augmenting and maintaining the pancreatic progenitor pool, with an endocrine roadblock upon its loss, persistent NEUROG3 and SOX9 expression and increased cell death. We demonstrate that *RFX6* haploinsufficiency does not affect beta cell number or insulin content but does impair function, predisposing carriers to diabetes. Our allelic series isogenic SC-islet models represent a powerful tool to elucidate specific aetiologies of diabetes in humans, enabling the sensitive detection of aberrations in both beta cell development and function.

### Supplementary Information

Below is the link to the electronic supplementary material.

ESM (PDF 86890 KB)

## Data Availability

Ultra-deep bulk RNA-seq data for pancreatic differentiation stages 3, 5 and 7 of H1 *RFX6* genotypes are deposited in the Gene Expression Omnibus database with accession code GSE234289. Original western blot images are deposited at Mendeley (https://data.mendeley.com/datasets/g75drr3mgw/2). Any additional information required to reanalyse the data reported in this paper is available from the corresponding authors on request.

## Abbreviations

[Ca^2+^]_i_: Cytoplasmic Ca^2+^ concentration
CHGA: Chromogranin A
FDR: False discovery rate
GIP: Glucose-dependent insulinotropic polypeptide
GLP-1: Glucagon-like peptide 1
GSIS: Glucose-stimulated insulin secretion
hESC: Human embryonic stem cell
hMITO: Human-specific mitochondrial antibody
iPSC: Induced pluripotent stem cell
NEUROG3: Neurogenin 3
NKX6.1: NK6 homeobox 1
NMD: Nonsense-mediated mRNA decay
NSG: NOD-SCID-Gamma
PDX1: Pancreatic and duodenal homeobox 1
PP: Pancreatic polypeptide
RFX6: Regulatory factor X 6
SC-islets: Stem-cell-derived islets
SLC18A1: Solute carrier family 18 member A1
SOX9: SRY-box transcription factor 9
TIRF: Total internal reflection fluorescence
VDCC: Voltage-dependent calcium channel
WT: Wild-type
