# Supplementary material for: RFX6 haploinsufficiency predisposes to diabetes through impaired beta cell function
Source: Diabetologia. 2024 May 14;67(8):1642–62. doi: 10.1007/s00125-024-06163-y (PMC11343796; doi:10.1007/s00125-024-06163-y)
Supplement: Supplementary file 1 — ESM (PDF 86890 KB) [file 125_2024_6163_MOESM1_ESM.pdf]

Electronic supplementary material (ESM)

RFX6 haploinsufficiency predisposes to diabetes through impaired beta cell function

Hazem Ibrahim et al.

Corresponding authors

Hazem Ibrahim
E-mail: hazem.ibrahim@helsinki.fi
Timo Otonkoski
E-mail: timo.otonkoski@helsinki.fi

This PDF file includes:

ESM Methods
ESM Tables 1 to 6
ESM Figures 1 to 13

ESM Methods

Tools and software used in bulk RNA-seq analysis

Fastq filtering: cutadapt (version 2.6)

Mapping / alignment software: STAR (version 2.7.6a)

RNA-seq analysis software: R (version 4.1) using Bioconductor (version 3.14)

Read counting: Rsubread (version 2.8.2)

Venn diagram: ggvenn (version 0.1.8) https://github.com/yanlinlin82/ggvenn

Heatmap figures: ComplexHeatmap (version 2.10.0)

Volcano plot, Boxplot, and PCA figures: ggplot2 (version 3.3.5)

Genome annotations: GENCODE GRCh38.p13

RNA-seq adapter sequences:

5′-AGATCGGAAGAGCACACGTCTGAACTCCAGTCA-3′ (R1),

5′-AGATCGGAAGAGCGTCGTGTAGGGAAAGAGTGT-3′ (R2)

ESM Table 1 Primers for PCR, CRISPR off-targets and NGS sequencing

Primer	Sequence
RFX6 Fw	GGCAGTGTTAAAAGGATGCTTTGG
RFX6 Rv	GGGTGCCTAATTCTGCTTTCTG
RFX6 dsDNA Mutation TseI 200bp Fw	TATTATATATACTTTCATGTTCTGTTCTTAATGAGTTCATGTTAAAGAAAAAAATCTTAACATACTTTTTACTCTGCAACTAGATCCAGCATTTTTTGCTGCTTTTGG
RFX6 dsDNA Mutation TseI 200bp Rv	TACCTTATAAAGAATTGAGTCACAAACACAGAAAATATCAATGATAACAGGATTTTCCAATAAGGGAAGCAAGTGATCAGGCATTCCTTGCCAAAAGCAGCAAAAAAT
RFX6 Cpf1 Off-target1 Fw	TCCCAGGATTAAGTGTGGGG
RFX6 Cpf1 Off-target1 Rv	TCAATATTTGGTACCTGCCAACT
RFX6 Cpf1 Off-target2 Fw	TCCTGCACAGAGGTCCTACA
RFX6 Cpf1 Off-target2 Rv	AGCATCATGGGTGTTCGACT
RFX6 Cpf1 Off-target3 Fw	CAGGGAACGAGGGAGAACTG
RFX6 Cpf1 Off-target3 Rv	GAGTTTCCTAGGTGGGTGCC
RFX6 cDNA NGSseq Fw	*ACACTCTTTCCCTACACGACGCTCTTCCGATCT*ACTCTGCAACTAGATCCAGCATTTT
RFX6 cDNA NGSseq Rv	*AGACGTGTGCTCTTCCGATCT*AAGAATTGAGTCACAAACACAGAAA

ESM Table 2 Differentiation protocol and media formulations for hESCs

Stage	Reagent	Medium
Stage 1<br>3 days	d0: 100 ng/mL human Activin A, 3 µmol/l CHIR<br>d1: 100 ng/ml human Activin A, 0.3 µmol/l CHIR<br>d2: 100 ng/ml human Activin A	Basal 1: MCDB131 (10372-019, Life Technologies), 2 mmol/l Glutamax, 1.5 g/l NaHCO3 (Sigma-Aldrich), 0.5% BSA fraction V Fatty acid free (Sigma-Aldrich), 10 mmol/l glucose
Stage 2<br>3 days	0.25 mmol/l Ascorbic acid, 50 ng/ml FGF7	
Stage 3<br>2 days	0.25 mmol/l Ascorbic acid, 50 ng/ml FGF7, 0.25 µmol/l SANT1, 1 µmol/l Retinoic Acid, 100 nmol/l LDN and 200 nmol/l TPB	Basal 2: MCDB131, 2 mmol/l Glutamax, 2.5 g/l NaHCO3, 2% BSA fV, 10 mmol/l glucose, 1:200 ITS-X (51500-056, Life Technologies)
Stage 4<br>4 days	0.25 mmol/l Ascorbic acid, 2 ng/ml FGF7, 0.25 µmol/l SANT1, 0.1 µmol/l Retinoic Acid, 200 nmol/l LDN, 100 ng/ml EGF, 10 mmol/l Nicotinamide, 100 nmol/l TPB and 10 µmol/l ROCKi	
Stage 5<br>4 days	0.25 µmol/l SANT1, 0.05 µmol/l Retinoic acid, 100 nmol/l LDN, 10 µmol/l ALK5inhII, 1 µmol/l GC1, 20 ng/ml Betacellulin, 100 nmol/l GSiXX and 5 µmol/l ROCKi	Basal 3: MCDB131, 2 mmol/l Glutamax, 1.5 g/l NaHCO3, 2% BSA fV, 20 mmol/l glucose, 1:200 ITS-X, 10 µg/ml Heparin (H3149, Sigma-Aldrich), 10 µmol/l Zinc Sulfate
Stage 6<br>8 days	100 nmol/l LDN, 10 µmol/l ALK5inhII, 1 µmol/l GC1 and 100 nmol/l GSiXX	
Stage 7<br>21 days	1 mmol/l N-Acetylcysteine, 10 nmol/l Tri-iodothyronine (T3) and 0.5 µmol/l Aurora kinase inhibitor ZM447439	CMRL-modified: CMRL 1066 (15-110-CVR, Corning), 2% BSA fV, 2 mmol/l Glutamax, 1:200 ITS-X, Heparin 10 µg/ml, 10 µmol/l Zinc Sulfate, 5 mmol/l Sodium Pyruvate (Lonza), 1:2000 chemically defined lipid concentrate, 1:2000 medium trace elements A, 1:2000 medium trace elements B

ESM Table 3 Differentiation protocol and media formulations for iPSCs

Stage	Reagent	Medium
Stage 1<br>2 days	d0: 100 ng/ml human Activin A, 3 µmol/l CHIR<br>d1: 100 ng/ml human Activin A, 0.3 µmol/l CHIR	Basal 1: MCDB131 (10372-019, Life Technologies), 2 mmol/l Glutamax, 1.5 g/l NaHCO3 (Sigma-Aldrich), 0.5% BSA fraction V Fatty acid free (Sigma-Aldrich), 10 mmol/l glucose
Stage 2<br>3 days	0.25 mmol/l Ascorbic acid, 50 ng/ml FGF7	
Stage 3<br>2 days	0.25 mmol/l Ascorbic acid, 50 ng/ml FGF7, 0.25 µmol/l SANT1, 1 µmol/l Retinoic Acid, 100 nmol/l LDN and 200 nmol/l TPB	Basal 2: MCDB131, 2 mmol/l Glutamax, 2.5 g/l NaHCO3, 2% BSA fV, 10 mmol/l glucose, 1:200 ITS-X (51500-056, Life Technologies)
Stage 4<br>4 days	0.25 mmol/l Ascorbic acid, 2 ng/ml FGF7, 0.25 µmol/l SANT1, 0.1 µmol/l Retinoic Acid, 200 nmol/l LDN, 100 ng/ml EGF, 100 nmol/l TPB and 10 mmol/l Nicotinamide	
Stage 5<br>4 days	0.25 µmol/l SANT1, 0.05 µmol/l Retinoic acid, 100 nmol/l LDN, 10 µmol/l ALK5inhII, 1 µmol/l GC1, 20 ng/ml Betacellulin and 100 nmol/l GSiXX	Basal 3: MCDB131, 2 mmol/l Glutamax, 1.5 g/l NaHCO3, 2% BSA fV, 20 mmol/l glucose, 1:200 ITS-X, 10 µg/ml Heparin (H3149, Sigma-Aldrich), 10 µmol/l Zinc Sulfate
Stage 6<br>12 days	100 nmol/l LDN, 10 µmol/l ALK5inhII, 1 µmol/l GC1, 100 nmol/l GSiXX and 10 µmol/l ROCKi	

ESM Table 4 Differentiation growth factors and reagents

Chemical/Reagent	Company name	Catalog#
ROCKi Y-27632	Selleckchem; Houston, USA	Cat# S1049
Activin A	QKine Ltd; Cambridge, UK	Cat# Qk001
CHIR	Tocris; Bristol, UK	Cat# 4423
Ascorbic acid	Sigma-Aldrich; USA	Cat# A4544
FGF7	Genscript; USA	Cat# Z03047
SANT1	Sigma-Aldrich	Cat# 4572
LDN	Selleckchem	Cat# S2618
Retinoic acid	Sigma-Aldrich	Cat# R2625
TPB	Santa Cruz; USA	Cat# sc-204424
EGF	Peprotech; USA	Cat# AF-100-15
Nicotinamide	Sigma-Aldrich	Cat# N0636
ALK5inhII	Selleckchem	Cat# S7223
GC1	Tocris	Cat# 4554
GSiXX	Millipore; Burlington, USA	Cat# 56578
N-Acetylcysteine	Sigma-Aldrich	Cat# A9165
Tri-iodothyronine (T3)	Sigma-Aldrich	Cat# T6397
Aurora kinase inhibitor ZM447439	Selleckchem	Cat# S1103
Glutamax	Life Technologies; USA	Cat# 35050038
Heparin	Sigma-Aldrich	Cat# H3149
Zinc Sulfate	Sigma-Aldrich	Cat# Z0251
Chemically defined lipid concentrate	Invitrogen; USA	Cat# 11905-031
Medium trace elements A	Corning; USA	Cat# 25-021-CI
Medium trace elements B	Corning	Cat# 99-175-CI
Cycloheximide	Sigma-Aldrich	Cat# 01810-1G
Exendin-4	Tocris	Cat# 1933

ESM Table 5 Antibodies used for immunofluorescence, flow cytometry and immunoblotting

Antibody	Company	Catalog#	Dilution
Rabbit anti-OCT4 Rabbit	Santa Cruz	Cat# sc-9081; RRID: AB_2167703	1:250 ICC
Rabbit anti-SSEA4 mAb DyLight™ 488	Thermo Fisher Scientific; USA	Cat# MA1-021-D488; RRID: AB_2536688	1:100 ICC
Mouse anti-TRA 1-60 mAb	Thermo Fisher Scientific	Cat# MA1-023; RRID: AB_2536699	1:100 ICC
Goat anti-PDX1	R&D systems; USA	Cat# AF2419; RRID: AB_355257	1:500 ICC, IHC
Mouse anti-NKX6.1	DSHB Hybridoma; USA	Cat# F55A10; RRID: AB_532378	1:250 ICC, IHC
Sheep anti Human Neurogenin-3	R&D systems	Cat# AF3444; RRID: AB_2149527	1:250 ICC, IHC
Rabbit anti-SOX9	Sigma-Aldrich	Cat# AB5535; RRID: AB_2239761	1:500 ICC, IHC
Mouse anti-GCG antibody	Sigma-Aldrich	Cat# G2654; RRID: AB_259852	FC-1:160 IHC- 1:500
Guinea pig anti-Insulin	Dako; USA	Cat# A0564; RRID: AB_10013624	1:500 ICC, IHC
Rabbit anti-Somatostatin	Dako	Cat# A0566; RRID: AB_2688022	1:500 ICC, IHC
Goat anti-Pancreatic polypeptide	Sigma-Aldrich	Cat# SAB2500747; RRID: AB_10611538	1:500 ICC, IHC
Goat anti-Ghrelin	Santa Cruz	Cat# sc-10368; RRID: AB_2232479	1:500 ICC, IHC
Mouse anti-CHGA	Dako	Cat# M0869; RRID: AB_2081135	FC-1:160 IHC- 1:500
Rabbit anti-SLC18A1	Sigma-Aldrich	Cat# HPA063797; RRID: AB_2685125	1:250 IHC
Mouse anti-human Mitochondria clone 113-1	Sigma-Aldrich	Cat# MAB1273; RRID: AB_94052	1:150 IHC
Mouse Anti-CD184 (CXCR4) Monoclonal Antibody	BD Biosciences; USA	Cat# 555974; RRID: AB_396267	1:10 FC
Mouse IgG2a, kappa Isotype Control, Phycoerythrin Conjugated	BD Biosciences	Cat# 5555749; RRID: AB_396091	1:10 FC
PE-Mouse anti PDX1	BD Biosciences	Cat# 562161; RRID: AB_10893589	1:80 FC
Alexa Fluor 647 Mouse anti NKX6-1	BD Biosciences	Cat# 563338; RRID: AB_2738144	1:80 FC
Alexa Fluor 647 Mouse IgG1 k isotype control	BD Biosciences	Cat# 557714; RRID: AB_396823	1:80 FC
Alexa Fluor 647 Rabbit anti Insulin (C27C9)	Cell Signaling Technology; USA	Cat# 9008; RRID: AB_2687822	1:80 FC
Alexa Fluor 647 Rabbit IgG Isotype Control	Cell Signaling Technology	Cat# 3452S; RRID: AB_10695811	1:80 FC

Alexa FluoR 488 Donkey anti-Mouse IgG secondary antibody	Thermo Fisher Scientific	Cat# A-21202; RRID: AB_141607	1:500
Alexa FluoR 594 Goat anti-Guinea Pig IgG secondary antibody	Thermo Fisher Scientific	Cat# A-11076; RRID: AB_2534120	1:500
Alexa FluoR 594 Donkey anti-Sheep IgG secondary antibody	Thermo Fisher Scientific	Cat# A- 11016; RRID: AB_2534083	1:500
Alexa FluoR 488 Donkey anti-Rabbit IgG secondary antibody	Thermo Fisher Scientific	Cat# A-21206; RRID: AB_2535792	1:500
Alexa FluoR 488 Donkey anti-Mouse IgG secondary antibody	Thermo Fisher Scientific	Cat# A-21203; RRID: AB_141633	1:500
Alexa FluoR 488 Donkey anti-Goat IgG secondary antibody	Thermo Fisher Scientific	Cat# A-11055; RRID: AB_2534102	1:500
Mouse anti-alpha-Tubulin Mouse mAb	Sigma-Aldrich	Cat# T5168; (B-5-1-2); RRID: AB_477579	1:2000 WB
Mouse anti-β-actin-HRP mAb	Santa Cruz	Cat# sc-47778; (C4); RRID: AB_626632	1:1000 WB
Rabbit anti-RFX6	Sigma-Aldrich	Cat# HPA037696; RRID: AB_2675616	1:100 WB
Anti-rabbit IgG, HRP-linked Antibody	Cell Signaling Technology	Cat# 7074; RRID: AB_2099233	1:2000 WB
Anti-mouse IgG, HRP-linked Antibody	Cell Signaling Technology	Cat# 7076; RRID: AB_330924	1:5000 WB

ESM Table 6 Primers for quantitative RT-PCR

Primer	NCBI Reference	Sequence and amplicon size
PPIG primer pair Housekeeping gene	NM_004792	Fw: TCTTGTCAATGGCCAACAGAG Rv: GCCCATCTAAATGAGGAGTTG; 84 bp
OCT4 primer pair	NM_002701	Fw: TTGGGCTCGAGAAGGATGTG Rv: TCCTCTCGTTGTGCATAGTCG; 91 bp
SOX2 primer pair	NM_003106	Fw: GCCCTGCAGTACAACTCCAT Rv: TGCCCTGCTGCGAGTAGGA; 85 bp
NANOG primer pair	NM_024865.2	Fw: CTCAGCCTCCAGCAGATGC Rv: TAGATTTCATTCTCTGGTTCTGG; 94 bp
RFX6 primer pair	NM_173560.1	Fw: GCATGTCGAACTCCAGTCCT Rv: GGAGTCCAGGGTTTCTTGAG; 114 bp
PDX1 primer pair	NM_000209.3	Fw: AAGTCTACCAAAGCTCACGCG Rv: CGTAGGCGCCGCCTGC; 52 bp
SOX9 primer pair	NM_000346	Fw: ATCAAGACGGAGCAGCTGAG Rv: GGCTGTAGTGTGGGAGGTTG; 100 bp
NEUROG3 primer pair	NM_020999	Fw: GACGACGCGAAGCTCACCAA Rv: TACAAGCTGTGGTCCGCTAT; 98 bp
INS primer pair	NM_000207	Fw: CAGAAGCGTGGCATTGTGGA Rv: GCTGCGTCTAGTTGCAGTAG; 82 bp
GCG primer pair	NM_002054	Fw: GAAGGCGAGATTTCCCAGAAG Rv: CCTGGCGGCAAGATTATCAAG; 113 bp
GHRL primer pair	NM_016362	Fw: TGAACACCAGAGAGTCCAGCA Rv: GCTTGGCTGGTGGCTTCTT; 53 bp
PPY primer pair	NM_002722	Fw: ATGCCACACCAGAGCAGATG Rv: CAGCGTGTCCTCTTTGTGTC; 104 bp
SST primer pair	NM_001048	Fw: CCCAGACTCCGTCAGTTTCT Rv: ACAGCAGCTCTGCCAAGAAG; 88 bp

ESM Fig. 1

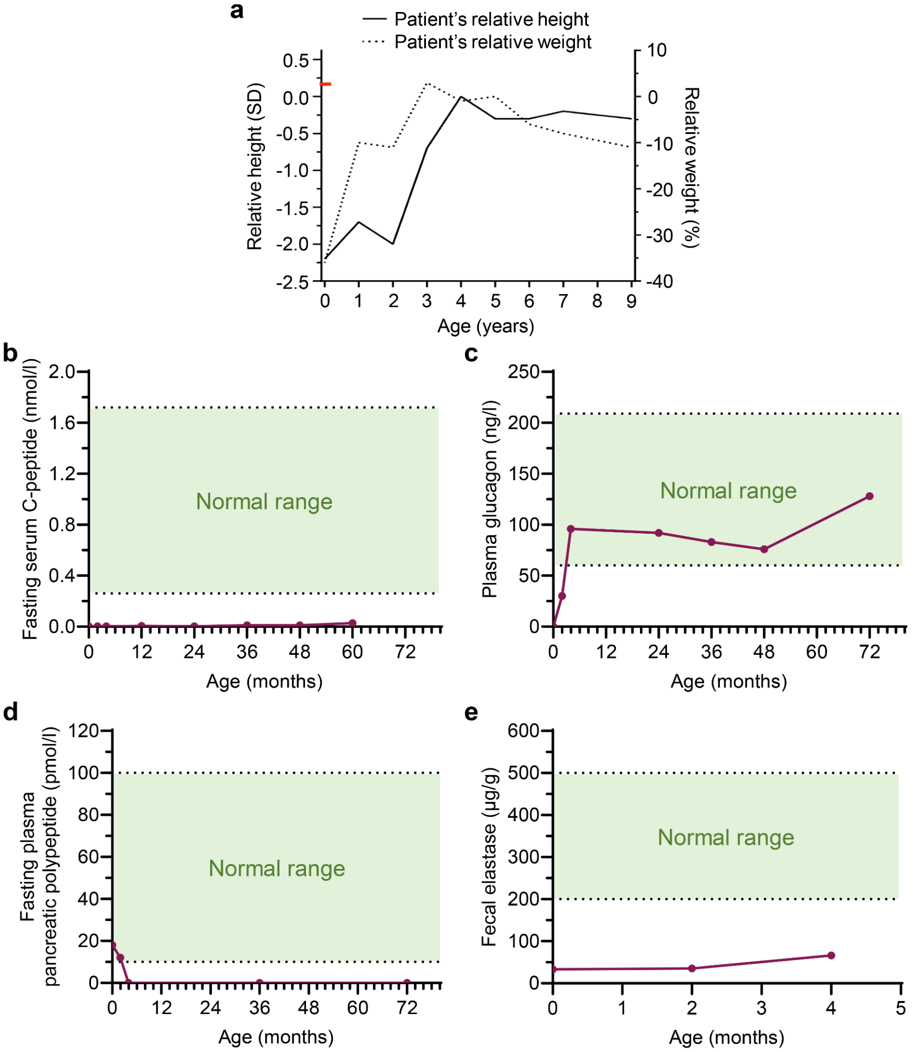

ESM Fig. 1 Clinical manifestations of the homozygous *RFX6* patient.

(**a**) Growth chart showing the patient's relative height and weight. Patient was born at gestation week 38+1, height; 47 cm and weight; 1800 g. Parental target relative height (+0.3 SD; red mark on left y axis). Relative height is depicted with solid line (left y axis) and relative weight (i.e. weight-for-height) with dotted line (right y axis).
(**b**) Fasting serum c-peptide levels in nmol/l during the first 5 years of age (normal range 0.26–1.72 nmol/l). Normal range in green represents clinical laboratory reference values.
(**c**) Plasma glucagon levels in ng/l during the first 6 years of age (normal range 60–209 ng/l).
(**d**) Fasting plasma pancreatic polypeptide levels in pmol/l during the first 6 years of age (normal range 10–100 pmol/l). Normal range in green represents clinical laboratory reference values.
(**e**) Fecal elastase levels in μg/g for the first 4 months after birth (normal level >200 μg/g). Normal range in green represents clinical laboratory reference values.

ESM Fig. 2

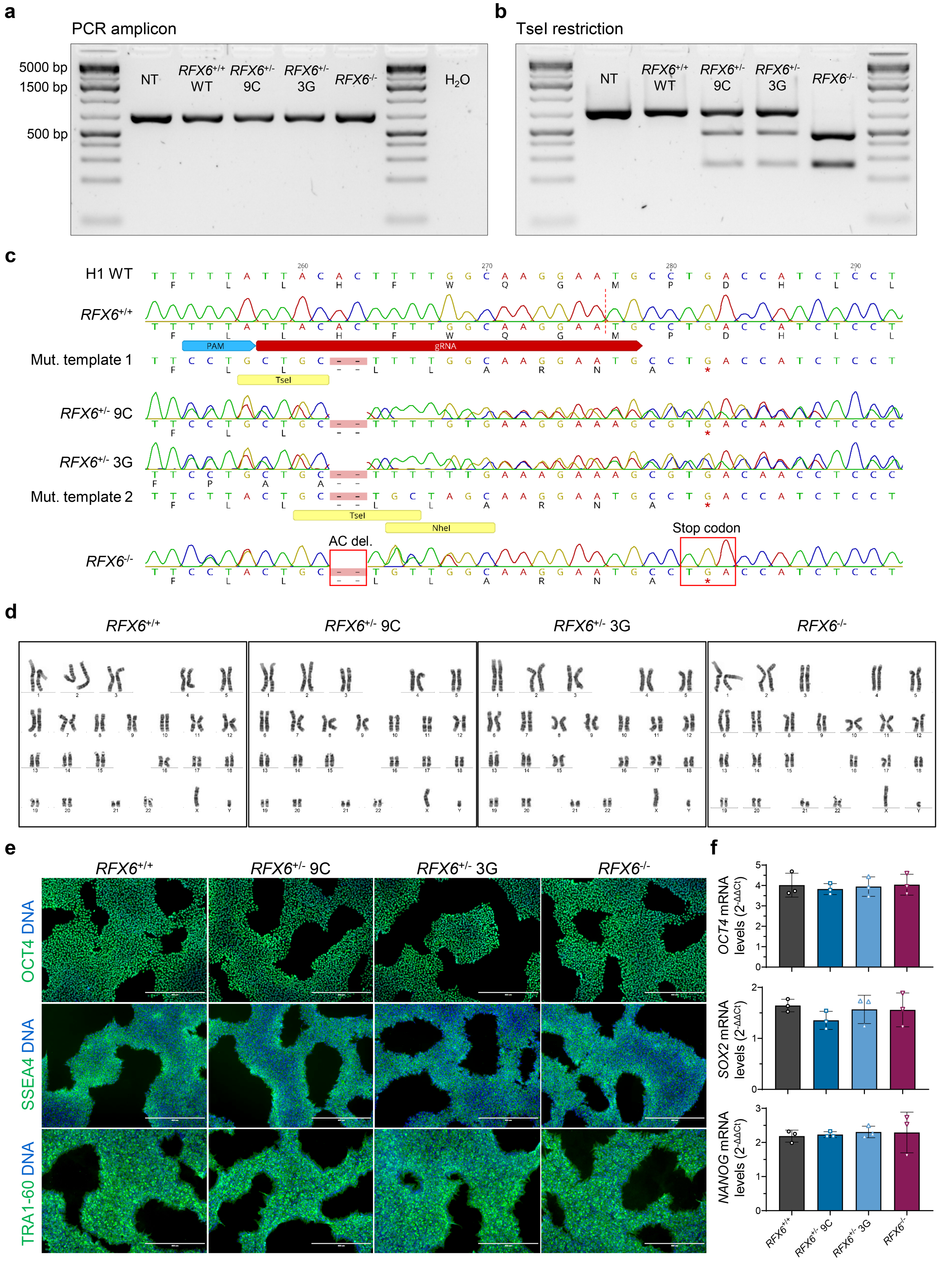
a
PCR amplicon
5000 bp
1500 bp
500 bp
NT
RFX6+/+ WT
RFX6+/- 9C
RFX6+/- 3G
RFX6-/-
H2O
b
TseI restriction
NT
RFX6+/+ WT
RFX6+/- 9C
RFX6+/- 3G
RFX6-/-
c
H1 WT
RFX6+/+
PAM
gRNA
Mut. template 1
TseI
RFX6+/- 9C
RFX6+/- 3G
Mut. template 2
TseI
NheI
AC del.
Stop codon
RFX6-/-
d
RFX6+/+
RFX6+/- 9C
RFX6+/- 3G
RFX6-/-
e
RFX6+/+
RFX6+/- 9C
RFX6+/- 3G
RFX6-/-
OCT4 DNA
SSEA4 DNA
TRA1-60 DNA
f
OCT4 mRNA levels (2-ΔΔCt)
SOX2 mRNA levels (2-ΔΔCt)
NANOG mRNA levels (2-ΔΔCt)
RFX6+/+
RFX6+/- 9C
RFX6+/- 3G
RFX6-/-

ESM Fig. 2 *RFX6* patient frameshift variant introduction in H1 hESCs using CRISPR-Cpf1.

(**a**) PCR gel electrophoresis showing 729 bp amplicons in all the cell-lines.
(**b**) PCR gel electrophoresis of the TseI restricted amplicons showing the restricted bands of the edited alleles 264+465 bp in heterozygous and homozygous cell-lines.
(**c**) Sanger sequencing showing the variant introduction heterozygously and homozygously using CRISPR-Cpf1 gRNAs and mutation templates.
(**d**) Normal karyotype visualised with G-banding for the four generated clones.
(**e**) Immunocytochemistry for pluripotency factors OCT4, TRA 1-60 and SSEA4 for the four clones. Scale bar, 400 µm.
(**f**) Relative gene expression levels of pluripotency factors *OCT4*, *SOX2* and *NANOG* for the four clones (n=3).
Statistical significance was measured using one-way ANOVA with Tukey's test for multiple comparisons correction in (f). Data are presented as means ± SD.

ESM Fig. 3

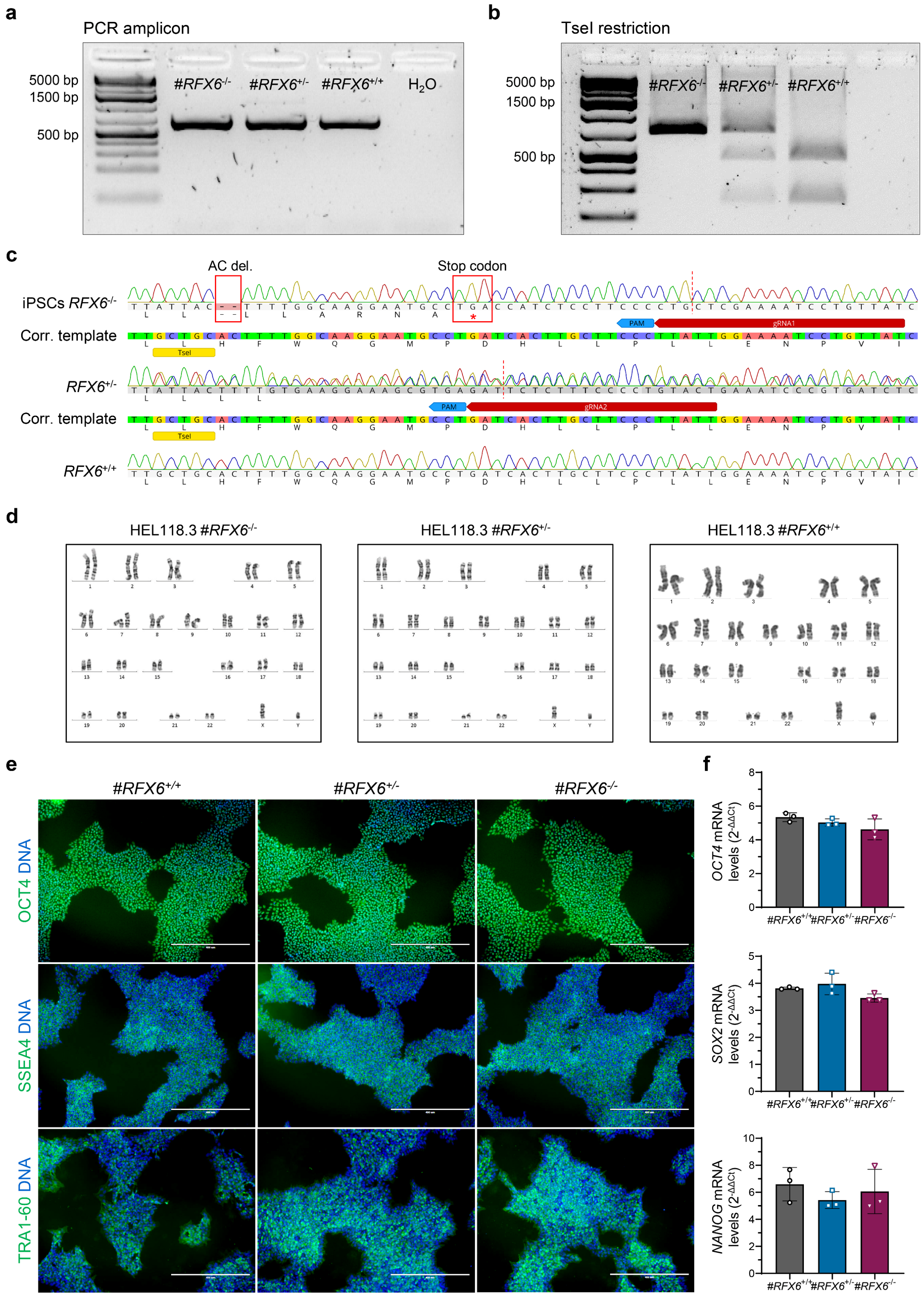
a
PCR amplicon
5000 bp
1500 bp
500 bp
#RFX6-/-
#RFX6+/-
#RFX6+/+
H2O
b
TseI restriction
5000 bp
1500 bp
500 bp
#RFX6-/-
#RFX6+/-
#RFX6+/+
c
AC del.
Stop codon
iPSCs RFX6-/-
Corr. template
RFX6+/-
Corr. template
RFX6+/+
PAM
gRNA1
gRNA2
TseI
d
HEL118.3 #RFX6-/-
HEL118.3 #RFX6+/-
HEL118.3 #RFX6+/+
e
#RFX6+/+
#RFX6+/-
#RFX6-/-
OCT4 DNA
SSEA4 DNA
TRA1-60 DNA
f
OCT4 mRNA levels (2-ΔΔCt)
SOX2 mRNA levels (2-ΔΔCt)
NANOG mRNA levels (2-ΔΔCt)
#RFX6+/+
#RFX6+/-
#RFX6-/-

ESM Fig. 3 *RFX6* frameshift variant correction in patient derived iPSCs using CRISPR-Cas9.

(**a**) PCR gel electrophoresis showing 729 bp amplicons of *RFX6* in all the cell-lines.
(**b**) PCR gel electrophoresis of the TseI restricted amplicons showing the restricted bands of the corrected alleles 264+465 bp in heterozygous and homozygous corrected cell-lines.
(**c**) Sanger sequencing showing the variant correction of the patient derived iPSCs using CRISPR-Cas9 gRNAs and a correction template.
(**d**) Normal karyotype visualised with G-banding for the three clones.
(**e**) Immunocytochemistry for pluripotency factors OCT4, TRA 1-60 and SSEA4 for the three clones. Scale bar, 400 µm.
(**f**) Relative gene expression levels of pluripotency factors *OCT4*, *SOX2* and *NANOG* for the three clones (n=3).
Statistical significance was measured using one-way ANOVA with Tukey's test for multiple comparisons correction in (f). Data are presented as means ± SD.

ESM Fig. 4

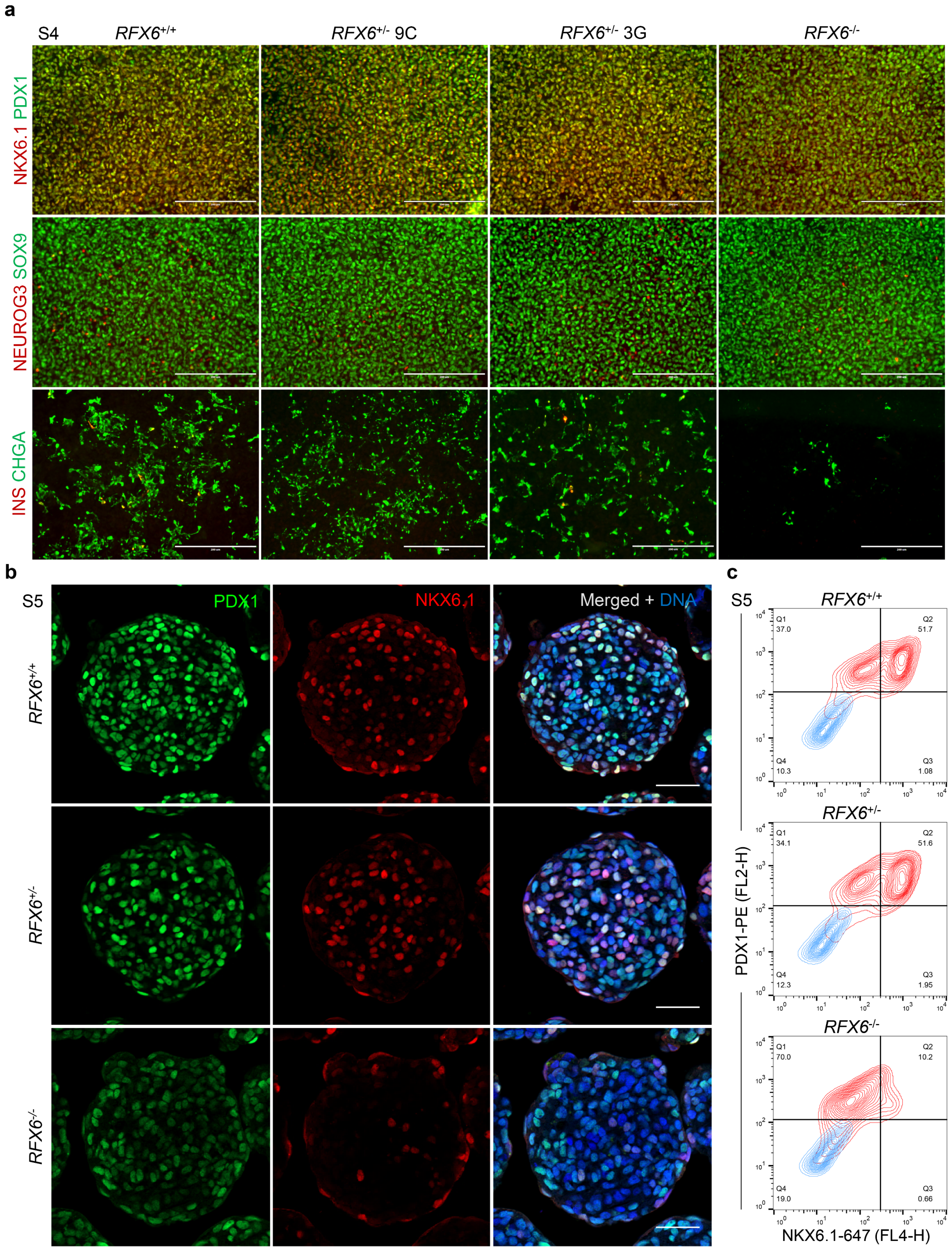

ESM Fig. 4 Reduced NKX6.1$^+$ cells at the endocrine precursor stage in *RFX6*$^{-/-}$.

(**a**) Immunocytochemistry showing PDX1$^+$ NKX6.1$^+$, SOX9$^+$ NEUROG3$^+$ and CHGA$^+$ INS$^+$ cells at S4. Scale bar, 200 μm.

(**b**) Immunohistochemistry showing PDX1$^+$ and NKX6.1$^+$ cells at S5. Scale bar, 50 μm.

(**c**) Flow cytometry analysis for PDX1$^+$ and NKX6.1$^+$ cells at S5. PDX1 and NKX6.1 sample is shown in red and IgG isotype negative control is shown in blue.

ESM Fig. 5

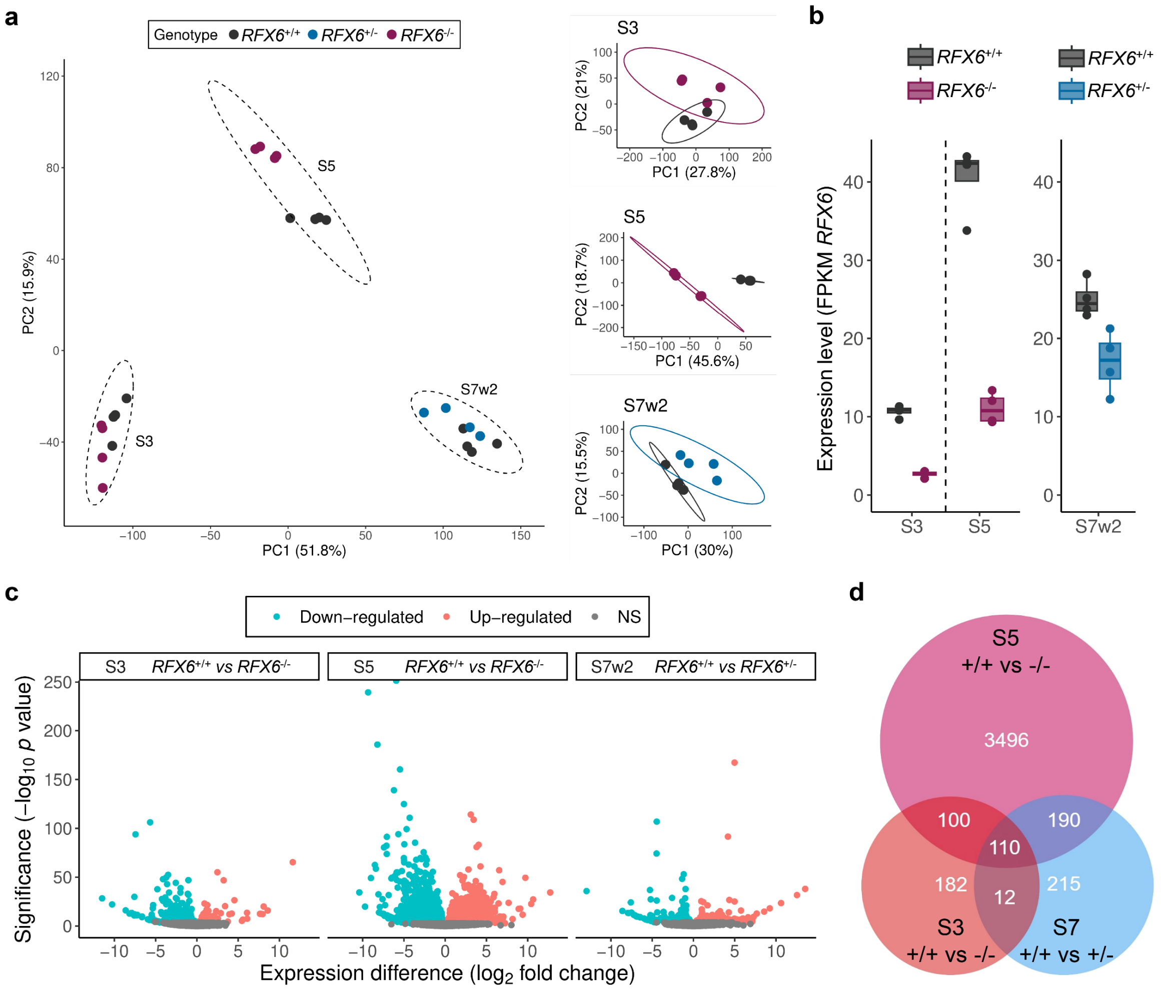

ESM Fig. 5 RNAseq analysis of H1 *RFX6* differentiated cells.

(**a**) Principal component analysis (PCA) of the RNA-seq data (using normalised read counts). The principal components that account for the majority of variation, separate the samples by developmental stage. The sub-plots show a PCA separately for each stage highlighting the differences between the genotypes; *RFX6*$^{+/+}$ vs *RFX6*$^{-/-}$ at S3 and S5, and *RFX6*$^{+/+}$ vs *RFX6*$^{+/-}$ at stage 7 week 2 (S7w2) (*n*=4).

(**b**) Boxplot of *RFX6* expression levels (FPKM) at different developmental stages for the tested genotypes (*n*=4).

(**c**) Volcano plots for differentially expressed genes for *RFX6*$^{+/+}$ vs *RFX6*$^{-/-}$ at S3 and S5, and for *RFX6*$^{+/+}$ vs *RFX6*$^{+/-}$ at S7w2. Significantly downregulated genes, iris blue; upregulated genes, soft red.

(**d**) Venn diagram showing the number and overlap of differentially expressed genes (FDR<0.01) between the genotypes within each developmental stage.

ESM Fig. 6

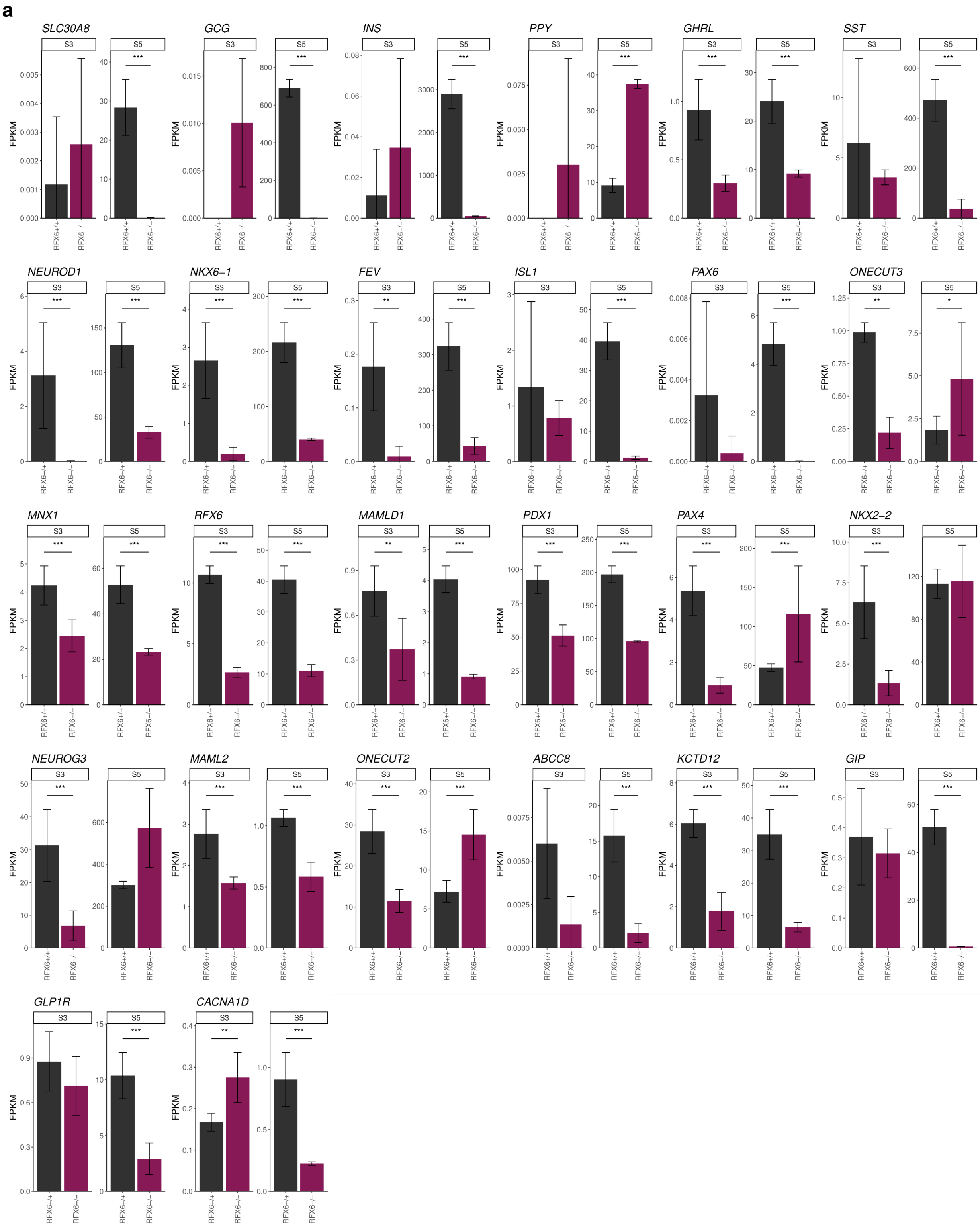

ESM Fig. 6 Reduced gene expression levels of pancreatic markers in *RFX6*$^{-/-}$.

(**a**) Barplots of average gene expression (FPKM) of the heatmap in (Fig. 2g). Statistical significance is indicated as False Discovery Rate corrected *p* value, *FDR<0.05, **FDR<0.01, ***FDR<0.001. Data are presented as means ± SD.

ESM Fig. 7

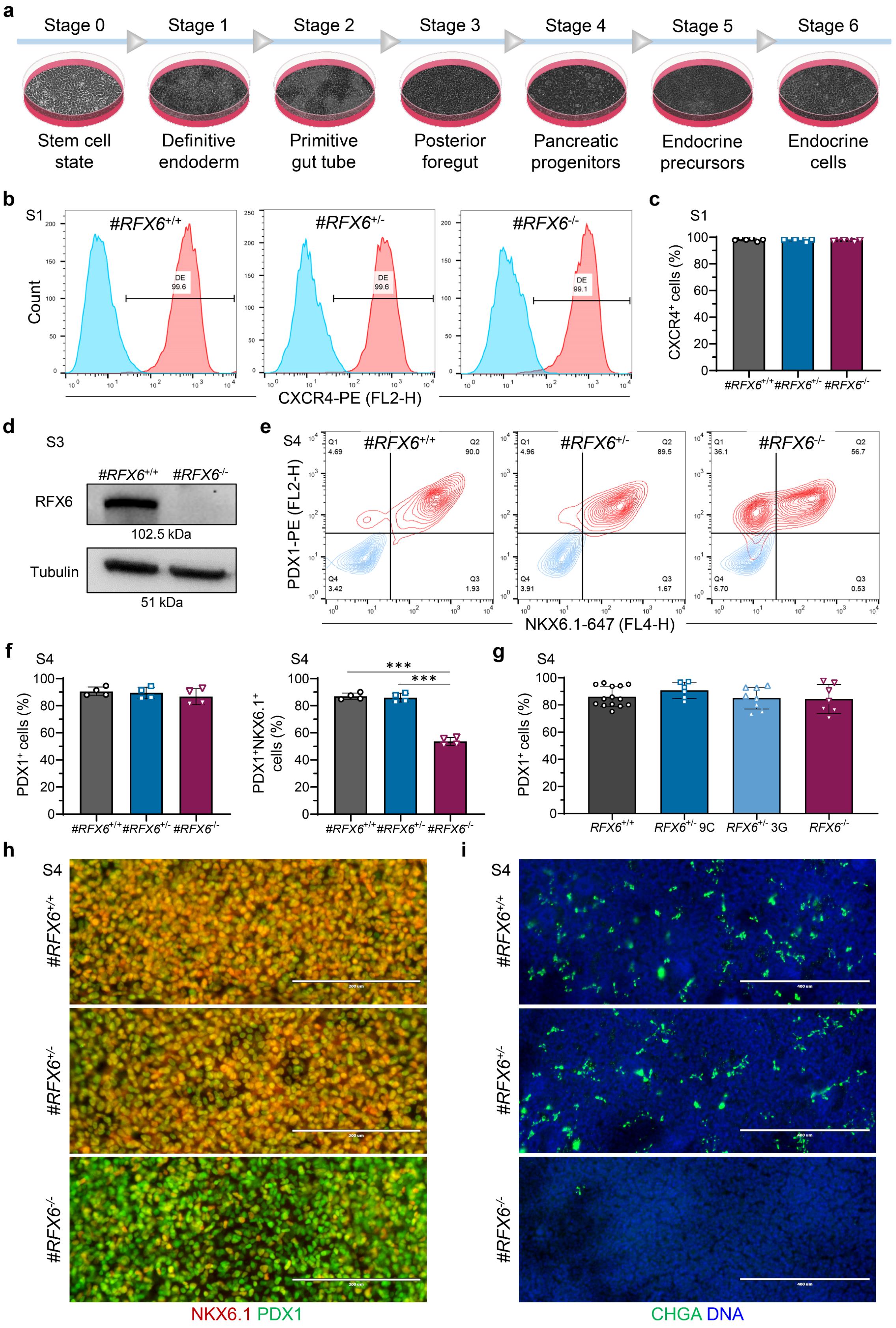
a
Stage 0
Stage 1
Stage 2
Stage 3
Stage 4
Stage 5
Stage 6
Stem cell state
Definitive endoderm
Primitive gut tube
Posterior foregut
Pancreatic progenitors
Endocrine precursors
Endocrine cells
b
S1
#RFX6+/+
#RFX6+/-
#RFX6-/-
Count
DE 99.6
DE 99.6
DE 99.1
CXCR4-PE (FL2-H)
c
S1
CXCR4+ cells (%)
#RFX6+/+ #RFX6+/- #RFX6-/-
d
S3
#RFX6+/+ #RFX6-/-
RFX6
102.5 kDa
Tubulin
51 kDa
e
S4
#RFX6+/+
#RFX6+/-
#RFX6-/-
PDX1-PE (FL2-H)
NKX6.1-647 (FL4-H)
Q1 4.69
Q2 90.0
Q4 3.42
Q3 1.93
Q1 4.96
Q2 89.5
Q4 3.91
Q3 1.67
Q1 36.1
Q2 56.7
Q4 6.70
Q3 0.53
f
S4
PDX1+ cells (%)
PDX1+NKX6.1+ cells (%)
***
***
#RFX6+/+ #RFX6+/- #RFX6-/-
g
S4
PDX1+ cells (%)
RFX6+/+
RFX6+/- 9C
RFX6+/- 3G
RFX6-/-
h
S4
#RFX6+/+
#RFX6+/-
#RFX6-/-
NKX6.1 PDX1
i
S4
#RFX6+/+
#RFX6+/-
#RFX6-/-
CHGA DNA

ESM Fig. 7 Reduced numbers of pancreatic progenitor cells in patient-derived iPSCs.

(**a**) Schematic of pancreatic endocrine differentiation protocol for 6 stages in monolayer.
(**b**) Flow cytometry analysis for the expression of C-X-C motif chemokine receptor 4-positive ($CXCR4^+$) definitive endoderm cells at the end of S1. CXCR4 sample is shown in red and IgG isotype negative control is shown in blue.
(**c**) Quantification of (b) (*n*=6).
(**d**) Immunoblot for RFX6 and α-Tubulin at S3 of control HEL46.11 (#$RFX6^{+/+}$) compared to the patient cell-line (#$RFX6^{-/-}$).
(**e**) Flow cytometry analysis for $PDX1^+$ and $NKX6.1^+$ cells in the iPSC-lines at S4. PDX1 and NKX6.1 sample is shown in red and IgG isotype negative control is shown in blue.
(**f**) Quantification of (e) (*n*=4).
(**g**) Flow cytometry quantification for $PDX1^+$ cells in the hESC-lines at S4 (*n*=7–14)
(**h**) Immunocytochemistry for $PDX1^+$ and $NKX6.1^+$ at the end of S4. Scale bar, 200 µm.
(**i**) Immunocytochemistry for $CHGA^+$ at the end of S4. Scale bar, 400 µm.
Statistical significance was measured using one-way ANOVA with Tukey's test for multiple comparisons correction in (c, f and g). Data are presented as means ± SD, $***p<0.001$.

ESM Fig. 8

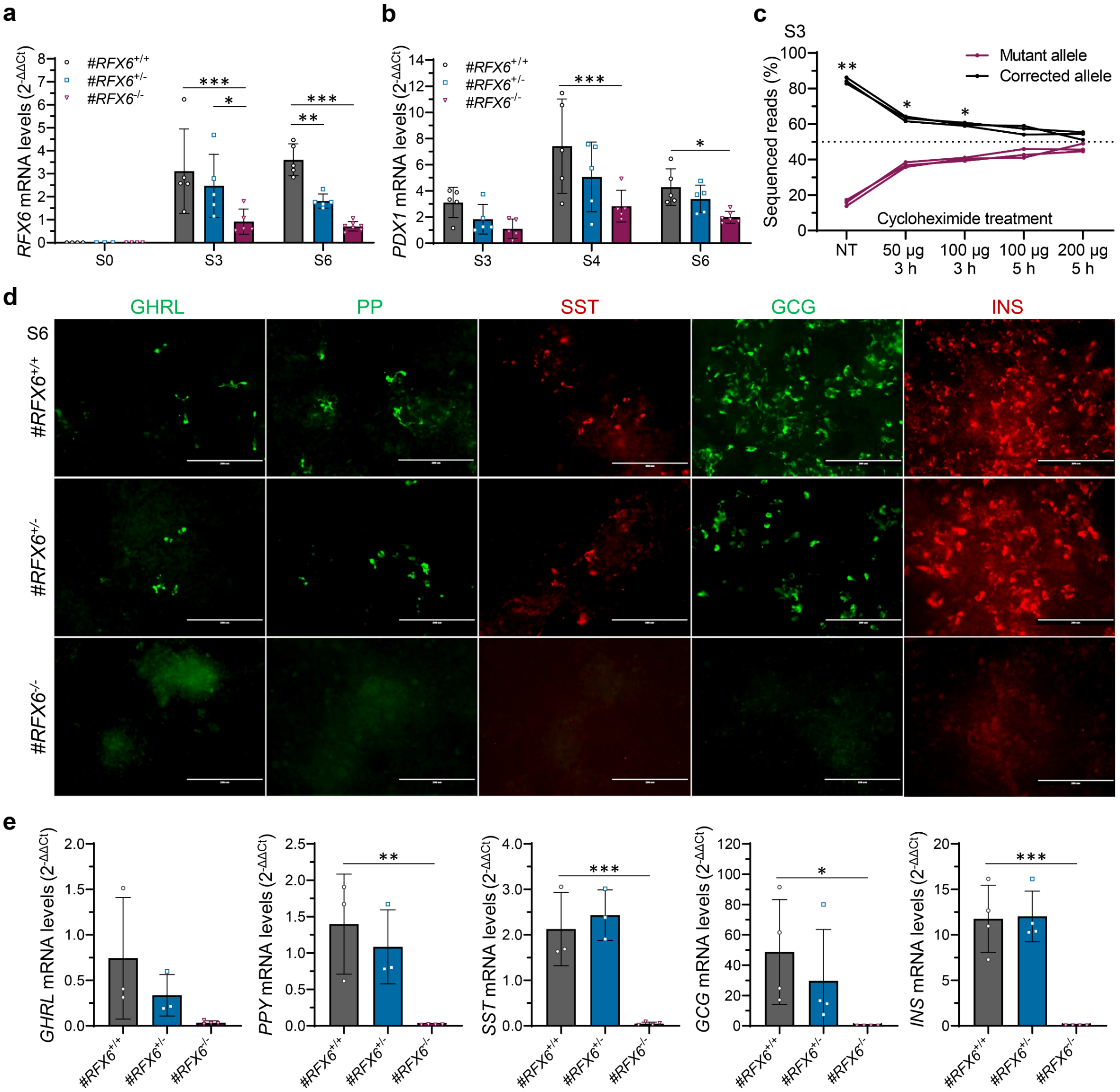

ESM Fig. 8 *RFX6* frameshift variant nonsense-mediated mRNA decay and diminished production of pancreatic endocrine cells from patient-derived iPSCs.

(**a**) Relative gene expression levels of *RFX6* at S0, S3 and S6 (*n*=3–7).
(**b**) Relative gene expression levels of *PDX1* at S3, S4 and S6 (*n*=5–6).
(**c**) Percentage of *RFX6* cDNA reads of the variant and corrected alleles at S3 of #*RFX6*$^{+/-}$ cell-line treated with different doses of the NMD inhibitor cycloheximide (*n*=3).
(**d**) Immunocytochemistry at S6 for the five pancreatic endocrine cell types. Ghrelin (GHRL), pancreatic polypeptide (PP), somatostatin (SST), glucagon (GCG) and insulin (INS). Scale bar, 200 µm.
(**e**) Relative gene expression levels of the five pancreatic hormones *GHRL*, *PPY*, *SST*, *GCG* and *INS* for the three isogenic cell-lines at S6 (*n*=3–4).
Statistical significance was measured using two-way ANOVA with Tukey's test for multiple comparisons correction in (a and b), using paired parametric multiple t-tests in (c), and using one-way ANOVA with Tukey's test for multiple comparisons correction in (e). Data are presented as means ± SD, *p<0.05, **p<0.01, ***p<0.001.

ESM Fig. 9

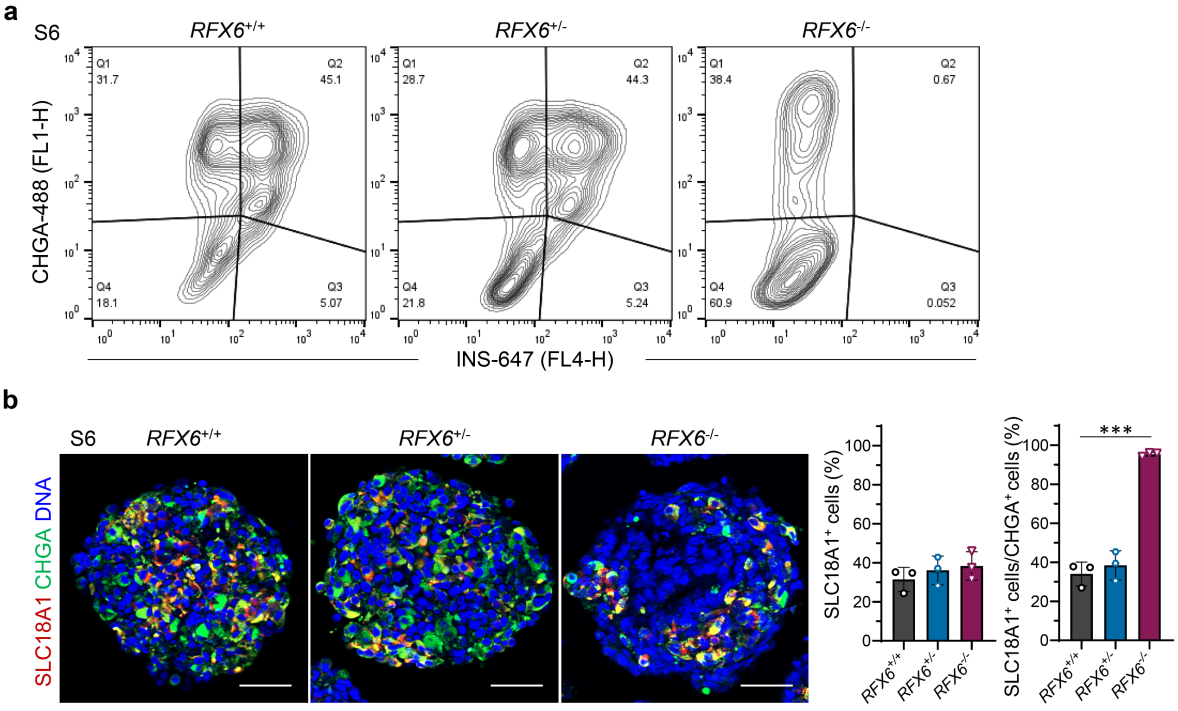

ESM Fig. 9 Reduced CHGA$^+$ endocrine cells in *RFX6*$^{-/-}$ at S6.

(**a**) Flow cytometry analysis for CHGA$^+$ cells (y-axis) and INS$^+$ cells (x-axis) at S6.
(**b**) Immunohistochemistry showing CHGA$^+$ (green) and SLC18A1$^+$ (red) cells in SC-islets at S6 (scale bar, 50 μm) and quantification of SLC18A1$^+$ cells per total cells and per CHGA$^+$ cells (n=3). Statistical significance was measured using one-way ANOVA with Tukey's test for multiple comparisons correction. Data are presented as means ± SD, ***p<0.001.

ESM Fig. 10

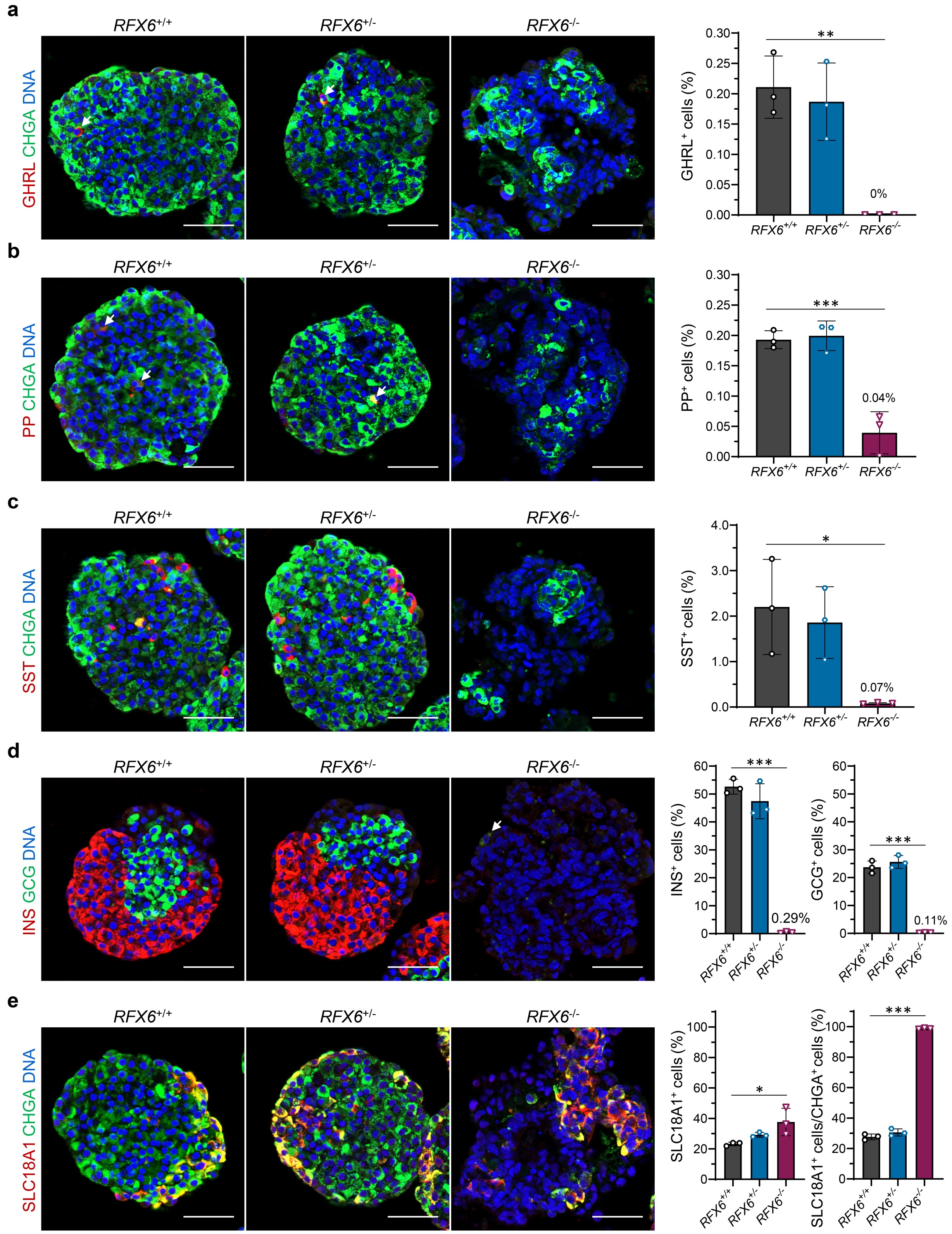
a
b
c
d
e
RFX6+/+
RFX6+/-
RFX6-/-
GHRL CHGA DNA
PP CHGA DNA
SST CHGA DNA
INS GCG DNA
SLC18A1 CHGA DNA
GHRL+ cells (%)
PP+ cells (%)
SST+ cells (%)
INS+ cells (%)
GCG+ cells (%)
SLC18A1+ cells (%)
SLC18A1+ cells/CHGA+ cells (%)
0%
0.04%
0.07%
0.29%
0.11%
**
***
*
***
***
*
***

ESM Fig. 10 Scarce pancreatic endocrine cells in *RFX6*$^{-/-}$.

(**a**) Immunohistochemistry showing CHGA^{+} (green) and GHRL^{+} (red with white arrows) cells in SC-islets at stage 7 week 2 (S7w2) for *RFX6*$^{+/+}$ and *RFX6*$^{+/-}$, and at stage 7 day 2 (S7d2) for *RFX6*$^{-/-}$ (scale bar, 50 µm), and quantification of GHRL^{+} cells (n=3).
(**b**) Immunohistochemistry showing CHGA^{+} (green) and PP^{+} (red with white arrows) cells in SC-islets at S7w2 for *RFX6*$^{+/+}$ and *RFX6*$^{+/-}$, and at S7d2 for *RFX6*$^{-/-}$ (scale bar, 50 µm), and quantification of PP^{+} cells (n=3).
(**c**) Immunohistochemistry showing CHGA^{+} (green) and SST^{+} (red) cells in SC-islets at S7w2 for *RFX6*$^{+/+}$ and *RFX6*$^{+/-}$, and at S7d2 for *RFX6*$^{-/-}$ (scale bar, 50 µm), and quantification of SST^{+} cells (n=3).
(**d**) Immunohistochemistry showing GCG^{+} (green) and INS^{+} (red) cells in SC-islets at S7w2 for *RFX6*$^{+/+}$ and *RFX6*$^{+/-}$, and at S7d2 for *RFX6*$^{-/-}$ (white arrow points at a GCG^{+} cell) (scale bar, 50 µm), and their quantification (n=3).
(**e**) Immunohistochemistry showing CHGA^{+} (green) and SLC18A1^{+} (red) cells in SC-islets at S7w2 for *RFX6*$^{+/+}$ and *RFX6*$^{+/-}$, and at S7d2 for *RFX6*$^{-/-}$ (scale bar, 50 µm), and quantification of SLC18A1^{+} cells per total cells and per CHGA^{+} cells (n=3).
Statistical significance in (a–e) was measured using one-way ANOVA with Tukey's test for multiple comparisons correction. Data are presented as means ± SD, *p<0.05, **p<0.01, ***p<0.001.

ESM Fig. 11

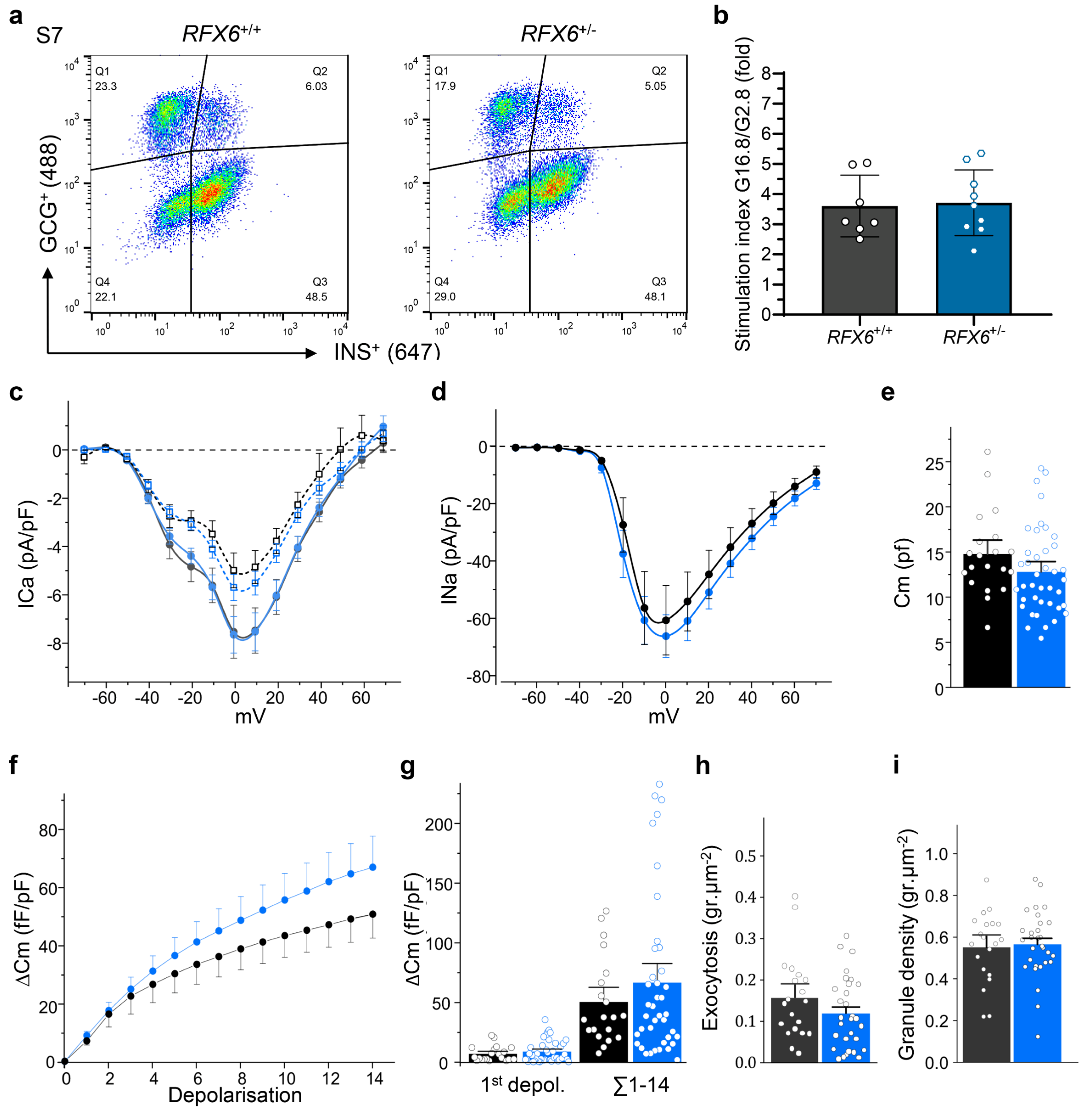

ESM Fig. 11 Voltage-dependence and exocytosis of *RFX6* SC-islets.

(**a**) Flow cytometry analysis for GCG^+ cells (y-axis) and INS^+ cells (x-axis) at stage 7 week 2 (S7w2).
(**b**) Insulin secretion stimulation index from static GSIS assays (G16.8 mmol/l / G2.8 mmol/l) (*n*=7–9).
(**c**) Voltage-dependence of Ca^{2+}-currents in *RFX6*$^{+/+}$ (black, *n*=20 cells) and *RFX6*$^{+/-}$ (blue, *n*=37 cells) beta cells, in absence (solid lines) or presence of 20 µM nifedipine.
(**d**) Voltage-dependence of Na^+-currents in the same cells as in (c).
(**e**) Initial whole cell capacitance, as measure of cell size.
(**f**) Exocytosis stimulated by a train of voltage-clamp depolarisations, measured as capacitance increase in *RFX6*$^{+/+}$ (black) and *RFX6*$^{+/-}$ (blue, *n*=40 cells) beta cells.
(**g**) Quantfication of capacitance changes in F during the first (left) and the sum of all depolarisations.
(**h-i**) Exocytosis (**h**) and initial docked granules (**i**) measured by TIRF microscopy of NPY-EGFP labelled insulin granules in *RFX6*$^{+/+}$ (black, *n*=20 cells) and *RFX6*$^{+/-}$ (blue, *n*=30 cells) beta cells. Cells were stimulated by elevating K^+ for 40s; data are normalised to cell footprint area.

ESM Fig. 12

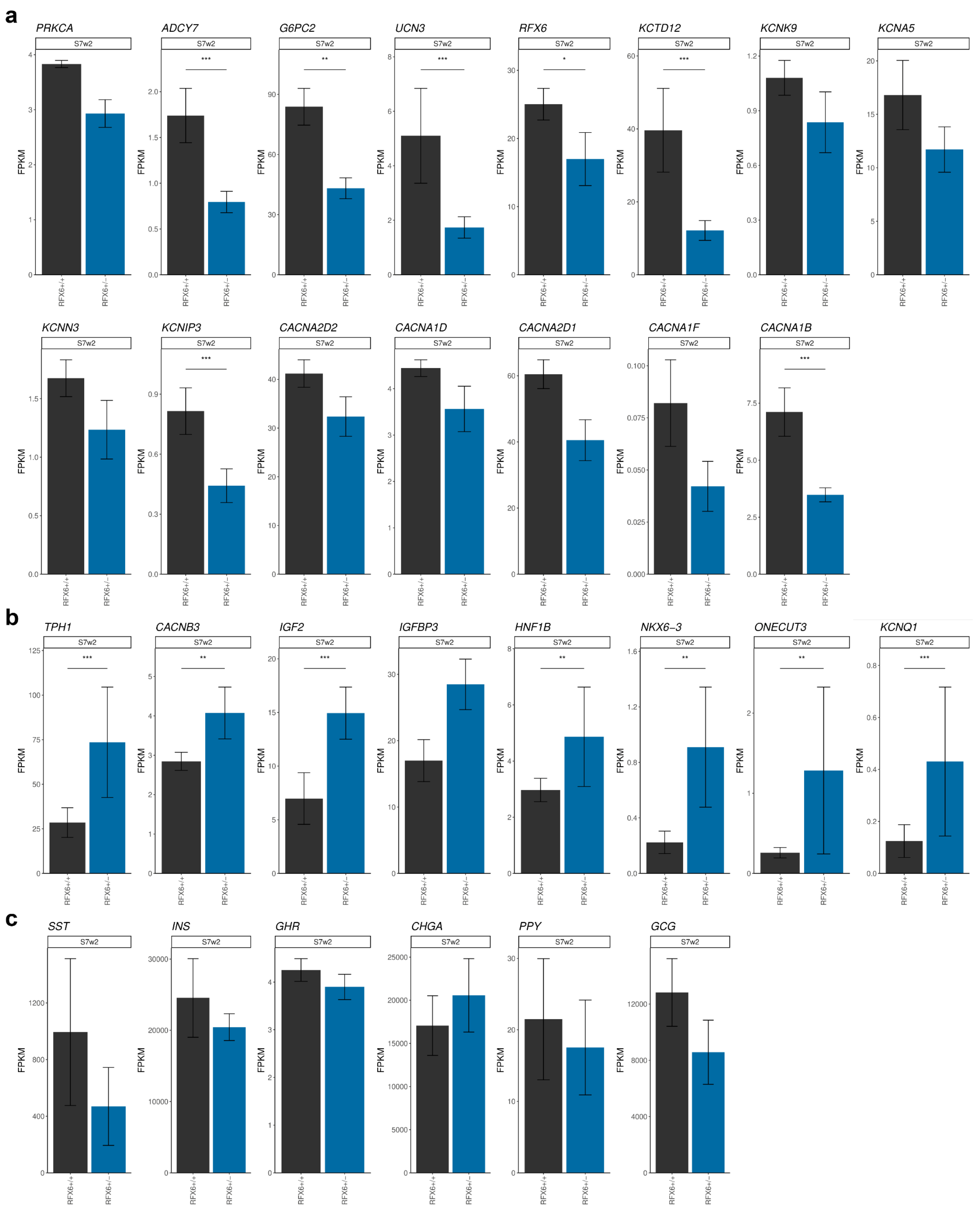

ESM Fig. 12 Altered gene expression levels in *RFX6*$^{+/-}$ at S7.

(**a**) Barplots of average gene expression (FPKM) of the upregulated genes heatmap in (Fig. 5c).
(**b**) Barplots of average gene expression (FPKM) of the downregulated genes heatmap in (Fig. 5d).
(**c**) Barplots of average gene expression (FPKM) of the hormone genes heatmap in (Fig. 5e).
Statistical significance is indicated as False Discovery Rate corrected *p* value, *FDR<0.05, **FDR<0.01, ***FDR<0.001. Data are presented as means ± SD.

ESM Fig. 13

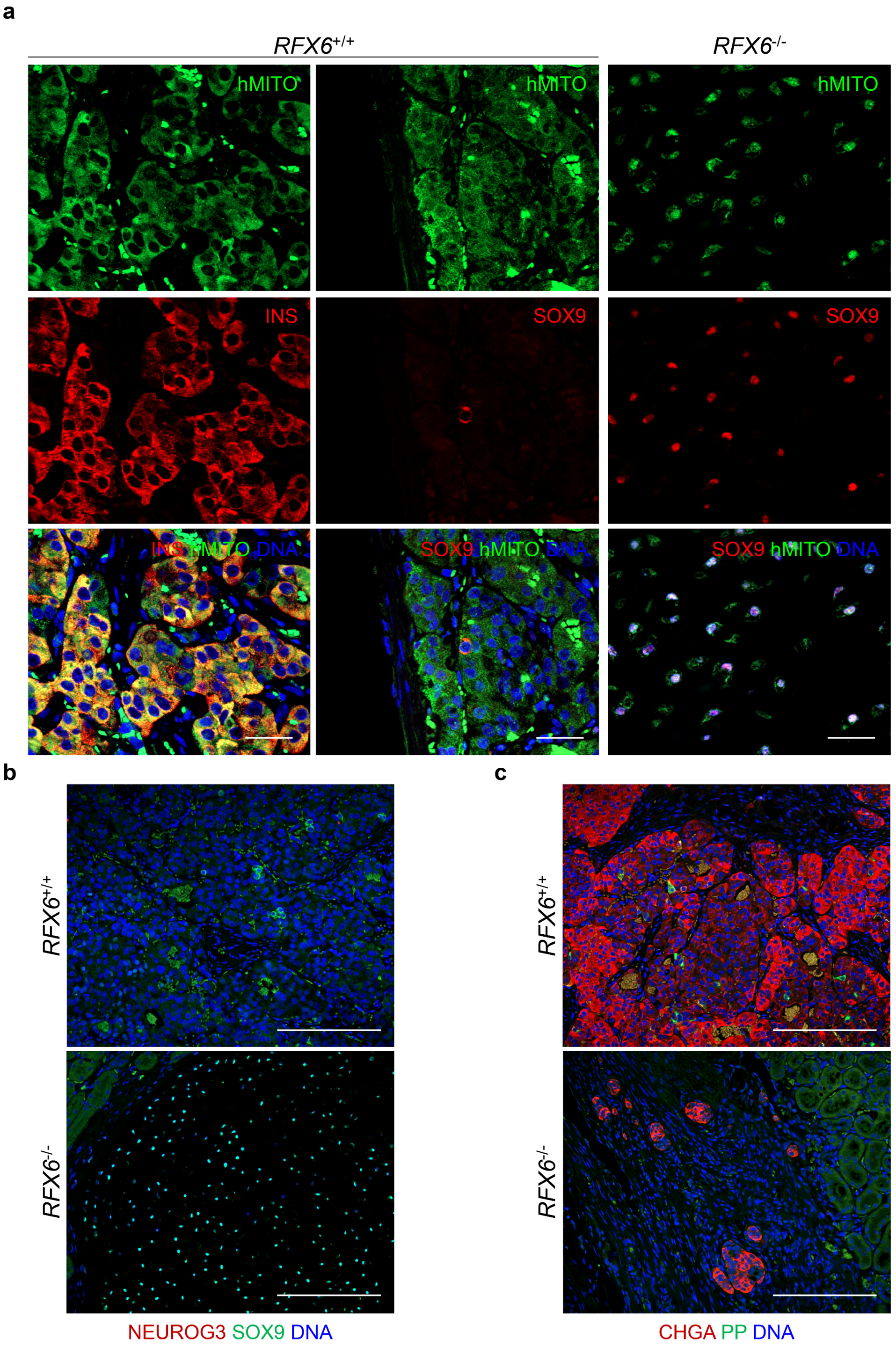

ESM Fig. 13 Persistent expression of SOX9 but not NEUROG3 in homozygous *RFX6*$^{-/-}$ cells post implantation.

(**a**) Immunohistochemistry showing human specific mitochondria (hMITO^{+}), INS^{+} and SOX9^{+} cells at month 3 post implantation. Scale bar, 50 μm.
(**b**) Immunohistochemistry showing SOX9^{+} and NEUROG3^{+} cells at month 3 post implantation. Scale bar, 200 μm.
(**c**) Immunohistochemistry showing PP^{+} and CHGA^{+} cells at month 3 post implantation. Scale bar, 200 μm.
